# HDAC7 controls anti-viral and anti-tumor immunity by CD8^+^ T cells

**DOI:** 10.3389/fimmu.2026.1816695

**Published:** 2026-05-12

**Authors:** Cansu Yerinde, Jacqueline Keye, Hsiang-Jung Hsiao, Sibel Durlanik, Inka Freise, Franziska Nowak, Marilena Letizia, Stephan Schlickeiser, Benedikt Obermayer, Adrian Huck, Marie Friedrich, Hao Wu, Désirée Kunkel, Anja A. Kühl, Sebastian Bauer, Andreas Thiel, Ahmed N. Hegazy, Britta Siegmund, Rainer Glauben, Carl Weidinger

**Affiliations:** 1 Medical Department, Division of Gastroenterology, Infectious Diseases and Rheumatology, Charité – Universitätsmedizin Berlin, corporate member of Freie Universität Berlin, Humboldt-Universität zu Berlin, Berlin, Germany; 2 Department of Chemistry, Biology, Pharmacy, Freie Universität Berlin, Berlin, Germany; 3 Flow & Mass Cytometry Core Facility, Berlin Institute of Health at Charité – Universitätsmedizin Berlin, Berlin, Germany; 4 BIH Center for Regenerative Therapies, Berlin Institute of Health at Charité – Universitätsmedizin Berlin, Berlin, Germany; 5 Core Unit Bioinformatics, Berlin Institute of Health at Charité – Universitätsmedizin Berlin, Berlin, Germany; 6 iPATH.Berlin-Immunopathology for Experimental Models, Core Facility of the Charité, Charité - Universitätsmedizin Berlin, Berlin, Germany; 7 Berlin University of Applied Sciences, Berlin, Germany; 8 Deutsches Rheuma-Forschungszentrum, ein Institut der Leibniz-Gemeinschaft, Berlin, Germany; 9 Clinician Scientist Program, BIH Academy, Berlin Institute of Health at Charité – Universitätsmedizin Berlin, Berlin, Germany; 10 German Cancer Consortium (DKTK), partner site Berlin, and German Cancer Research Center (DKFZ), Heidelberg, Germany

**Keywords:** apoptosis, CD8+ T cells, cellular exhaustion, colitis, Eomes, FasL, glutamine, HDAC7

## Abstract

Class II histone deacetylases (HDAC) orchestrate T cell-dependent immune responses via the epigenetic control of genes and via the post-translational modification of cytoplasmic and nuclear proteins. However, the contribution of single HDAC family members to the differentiation and function of peripheral CD8^+^ T cells remains elusive. We here demonstrate that HDAC7-deficiency leads to the upregulation of immune checkpoint molecules, increased apoptosis and disturbed glutamine homeostasis of peripheral murine CD8^+^ T cells, which we could link to a MEF2D-dependent induction of FasL expression ultimately deterring the survival of HDAC7-deficient CD8^+^ T cells. Likewise, we observed in mouse models of lymphoma, that mice with a T cell-specific deletion of *Hdac7* harbor impaired anti-tumor immune responses in syngeneic transfer models of lymphoma and we found that HDAC7 is required for CD8^+^ T cell-dependent memory recall responses in models of lymphocytic choriomeningitis virus infection. Taken together, we identify HDAC7 as a central regulator of cellular exhaustion and apoptosis of peripheral CD8^+^ T cells, controlling CD8^+^ T cell dependent anti-tumor and anti-viral immunity in mice.

## Introduction

CD8^+^ T cells are essential for anti-tumor and anti-viral immunity by killing neoplastic and infected cells and by providing long-term immunity through the generation of memory T (T_mem_) cells. Upon antigen stimulation, naïve CD8^+^ T cells rapidly expand and differentiate into short-lived cytotoxic T cells (CTL), identified as killer cell lectin-like receptor subfamily G member 1 (KLRG1)^+^ and CD127^-^ cells (1), which eradicate tumor target or virus-infected cells (2). Following pathogen clearance, the population of KLRG1^+^CD127^-^CD8^+^ T cells contracts via undergoing apoptosis and leaves behind a small population of antigen-specific, long-lived CD8^+^ memory T cells, commonly associated with a KLRG1^-^CD127^+^ phenotype, that can either rapidly give rise to new short-lived CTL, in case of antigen re-challenge (3), or endure years until antigen re-exposure, although recent studies suggest that subsets of KLRG1^+^ cells can retain functional plasticity (4).

Several mechanisms have been described to play a role in the differentiation and maintenance of memory and effector cells e.g., the strength of antigen stimulation or the expression of specific transcription factors like Eomesodermin (Eomes) in CD8^+^ T_mem_ and T-bet in CTL (5, 6). Recent work has also highlighted the significant metabolic changes CD8^+^ T cells must undergo during the effector to memory cell transition (7). Thus, high glycolytic activity favors the terminal differentiation and expansion of CTL, whereas decreased glycolytic flux enhances the formation of long-lived CD8^+^ T_mem_ cells (8). First evidence also suggests that several amino acids and amino acid transporters control the differentiation and function of CD8^+^ T_mem_ cells via the regulation of the mammalian target of rapamycin (mTOR) signaling (9). However, the pathways directing the metabolic switch in CD8^+^ T cells are not completely understood and it remains to be elucidated how these metabolic fate decisions are epigenetically controlled and imprinted in CD8^+^ T cells.

We have previously demonstrated that Store-operated Calcium Entry (SOCE) mediated by stromal interaction molecule (STIM)1, STIM2 as well as ORAI proteins, regulates effector functions of cytotoxic CD8^+^ T cells upon TCR activation such as the production of IFNy, TNFα as well as the degranulation of toxic vesicles (10, 11). Additionally, SOCE is required for the differentiation, maintenance and re-activation of CD8^+^ T_mem_ cells during anti-viral response in lymphocytic choriomeningitis virus (LCMV) infected *Stim1^fl/fl^Stim2^fl/fl^Cd4-Cre* mice (12). Importantly, Vaeth and colleagues previously showed that SOCE represents a central regulator in the metabolic switch of T cells, as SOCE-deficient T cells harbor a reduced activity of the Akt/mTOR pathway ultimately resulting in defective cellular expansion and differentiation upon antigen encounter (13). It is currently not known how SOCE signaling components are regulated and which epigenetic and post-translational modifications influence its signaling strength in T cells.

Continuous antigen stimulation can drive CD8^+^ T cells into a dysfunctional and exhausted state, which stands in contrast to functional effector or memory cells generated upon the resolution of infections (14). Thus, exhausted cells are incapable of clearing virus and cannot react with proper recall responses upon viral reinfection in mouse models of LCMV infections (14, 15). Besides chronic infections, T cell exhaustion was also found to play a central role in the immune evasion of tumors and still represents a major challenge in immune therapy of cancer (16). The main characteristics of cellular exhaustion include the increased expression of multiple inhibitory checkpoint molecules such as PD-1, TIM-3, CTLA-4 and LAG-3 together with a decreased production of the effector cytokines IL-2, IFNy and TNFα (17). Moreover, exhausted CD8^+^ T cells possess distinct epigenetic, transcriptional and metabolic profiles suggesting that they might represent a distinct subset of effector and memory CD8^+^ T cells (18–22). To date, the cellular signaling networks and epigenetic regulators of cellular exhaustion are incompletely understood.

Over the last years, the modification of histone acetylation patterns has been described in several studies as a key regulator of T cell differentiation and function (23–26). Among class II histone deacetylases (HDACs), HDAC7 has emerged as a prominent candidate that might not only control thymic development of T cells via the transcriptional regulation of apoptosis in thymic T cells (27–29), but which might also be pivotal for the function of adult peripheral T cells (30). Thereby, phosphorylation of HDAC7 and its subsequent nuclear export is observed upon TCR activation (31). Due to its function as nuclear and cytoplasmic deacetylase, HDAC7 can play dual roles, both as a transcriptional repressor as well as a regulator of protein-protein interactions via post-translational modifications of cytoplasmic target proteins and nuclear transcription factors such as the myocyte enhancer factor 2 (MEF2) (30–35). However, the functional role of HDAC7 in CD8^+^ T cell-mediated immune responses is so far unknown.

Here, we discovered that *Hdac7*-deficient CD8^+^ T cells display impaired SOCE as well as a deregulation of metabolism, exhaustion and apoptosis regulating genes, ultimately deterring metabolic fitness and survival of CD8^+^ T cells *in vitro*. By using conditional *Hdac7* knockout mice, we could furthermore demonstrate that the lack of HDAC7 results in dysfunctional CD8^+^ T cell-dependent anti-tumor immune responses due to a decreased survival of tumor-infiltrating CD8^+^ T cells paralleled by an increased cellular exhaustion of *Hdac7*-deficient CD8^+^ T cells *in vivo*. Moreover, we observed that HDAC7 is required for the formation of virus-specific memory CD8^+^ T cells and proper recall responses in LCMV models of infection, as *Hdac7-*deficient virus-specific CD8^+^ T cells featured an enrichment of exhaustion-related transcriptional signatures and failed to properly expand upon viral re-challenge models ultimately leading to uncontrolled viral replication in mice. Taken together, we here provide evidence that HDAC7 controls the cellular exhaustion and the survival of peripheral CD8^+^ T cells and that HDAC7 is required for proper anti-tumor and anti-viral immune responses.

## Materials and methods

### Mice

Six to twelve week-old *Hdac7^fl/fl^Cd4-Cre, Hdac7^fl/fl^E8I-Cre* or *Hdac7^fl/fl^Cd4-Cre-OTI-Thy1.1* mice were bred in the Charité animal facility under specific pathogen-free conditions. *CD45.1^+^* and *Rag2^-/-^*mice were 8 to 12 weeks old. For subsequent single cell analyses, mice were sacrificed and organs were harvested. All animal protocols were approved by the regional animal study committee of Berlin (LaGeSo, Berlin, Germany) and conducted accordingly.

### Stimulation and staining for mass cytometry

Splenocytes in suspension were stimulated for 5 h with 0.01 µg/ml phorbol myristate acetate (PMA, Sigma-Aldrich) and 1 µg/ml ionomycin (Sigma-Aldrich) in the presence of 0.01 mg/ml brefeldin A (Sigma-Aldrich). After stimulation, cells were stained with metal-conjugated antibodies for surface antigens and then incubated with 0.5 µM cisplatin (Fluidigm) to enable exclusion of dead cells. Afterwards, cells were fixed in 2% paraformaldehyde followed by permeabilization with saponin-based permeabilization buffer (Thermo Fisher Scientific). Intracellular cytokines were stained with metal-conjugated antibodies and the cell pellet was resuspended in nucleic acid intercalator-*Ir* solution (Fluidigm) for cell identification. Cells were acquired on a CyTOF2 mass cytometer upgraded to Helios specifications (CyTOF2/Helios; Fluidigm) as previously described (36). All metal-conjugated antibodies including the clone numbers and antibody dilutions are listed in **Supplementary Tables 1** and **2**.

### Gating and analysis for mass cytometry

Cytobank (Cytobank Inc.) was used for initial gating and embedded viSNE to generate t-distributed stochastic neighbor embedding (t-SNE) maps. To remove debris, doublets, normalization beads and erythrocytes the generated fcs-files were manually gated on cellular events by DNA intercalators, event length and TER119. For detailed T cell analysis, the resulting files were manually gated on CD45^+^TCRβ^+^CD3^+^ or CD45^+^TCRβ^+^CD3^+^CD8^+^ events. To generate 2-dimensional t-SNE maps the algorithm included following markers CD3, CD103, CD115, CD69, CD11b, CD25, Ly6G, Ly6C, MHCII, PD-L1, CD62L, CD83, PD-L2, CD8, TCRβ, CD44 and CD4 for the analysis of CD45^+^TCRβ^+^CD3^+^ T cells. IL-2, IFNγ, TNFα, CD40L and CD44 were included for t-SNE analysis of CD45^+^TCRβ^+^CD3^+^CD8^+^ events. Calculation of earth-mover’s distances between t-SNE maps was performed as previously described^1^.

### T cell transfer colitis experiments

*Rag2^-/-^* mice were intraperitoneally (i.p.) injected with 5x10^5^ naïve CD4^+^CD62L^+^CD44^-^ T cells from either Wt or *Hdac7^fl/fl^Cd4-Cre* mice to induce colitis. Body weight, stool consistency and rectal bleeding from transfer colitis mice were assessed every other day. Values assessed prior to T cell transfer served as baseline. Weight changes were calculated in relation to the weight at baseline (100%). Stool consistency was scored as follows: 0, well-formed pellets; 2, pasty and semi-formed stools; and 4, liquid stools. Rectal bleeding was scored using hemoccult tests (Beckman Coulter) as follows: 0, no blood; 2, positive hemoccult; and 4, macroscopically visible blood. Mice were sacrificed by cervical dislocation and the entire colon was removed and its length was determined. Colon sections were fixed in buffered formalin (10%) and embedded in paraffin. Sections were stained with hematoxylin & eosin and histological signs of inflammation were evaluated as a combined score ranging from 0–5 as previously described (37).

### Isolation and labeling of murine lamina propria mononuclear cells

The colon was trimmed of fat and mesenteric tissue and lamina propria mononuclear cells (LPMCs) were subsequently isolated as previously described^2^. The colon was opened longitudinally and cut into pieces of about 2 mm^2^. The tissue was incubated in calcium- and magnesium-free HBSS containing 1 mM EDTA for 30 min under vigorous vortexing at room temperature; this step was repeated once. Supernatants of the EDTA steps were discarded and the remaining tissue was washed twice and subsequently incubated for 90 min at 37 °C in calcium- and magnesium-free HBSS supplemented with 5% FCS and 100 U/ml collagenase D and 20 µg/ml DNAse I (both Sigma-Aldrich). Cells were separated from tissue debris by filtration through a 100 μm cell strainer (BD Pharmingen) followed by purification through a discontinuous 40/100% Percoll gradient (GE Healthcare) for 25 min at 1200 × *g*. Cells were subsequently incubated for 3 h in the presence of 10 µg/ml brefeldin A (Sigma-Aldrich), 7.5 ng/ml PMA and 1 µg/ml ionomycin or left unstimulated. For intracellular labeling, cells were stained in advance with LIVE/DEAD fixable aqua (LDaqua; Thermo Fisher Scientific) and then fixed for 5 min with 4% formalin. Cells were washed and intracellular staining was performed in 0.5% Saponin (Sigma) in PBS/BSA. For intranuclear staining, cells were processed using the FOXP3 transcription factor staining buffer kit (Thermo Fisher Scientific) according to the manufacturer’s protocol. Please also see **Supplementary Table 3** for information containing antibody dilutions and catalogue numbers.

### RNA sequencing

For RNA sequencing, RNA was isolated from flow-sorted (BD FACSAria Fusion, BD Bioscience) naïve (CD3^+^CD8^+^CD62L^+^CD44^-^), pre-activated (CD3^+^CD8^+^CD62L^+^CD44^+^), *in-vitro* activated (48 h after activation with anti-CD3/CD28) or LCMV-specific CD8^+^ T cells (day 8 p.i.) of *Hdac7^ko^* mice or Wt littermates using the Direct-zol RNA-MicroPrep kit (Zymo Research) according to the manufacturer’s protocol. Subsequently, RNA concentrations were determined using a Nanodrop spectral photometer (Thermo Fisher Scientific) and the integrity of RNA was assessed using a Tapestation 2200 (Agilent) following the manufacturer’s instructions. Samples with an RNA integrity number (RIN) of >8 were frozen at -80 °C and subsequently shipped to Microsynth AG Swiss for sequencing. The strand specific Illumina TruSeq RNA library preparation kit v2 including polyA enrichment was used to construct libraries from total RNA. Subsequently the Illumina NextSeq 500 platform and a 75 cycles high output v2 kit were used to sequence libraries. The produced single-end reads which passed Illumina’s chastity filter were subjected to de-multiplexing and trimming of Illumina adaptor residuals using Illumina’s real time analysis software (no further refinement or selection). Quality of the reads in fastq format was checked with the software FastQC (version 0.11.5).

### RNA sequencing analysis

A total of 24 fastq files (3 biologically independent samples per group and genotype) was obtained from RNA sequencing containing single-end reads. Reads were then aligned to the mouse genome using the STAR software package version 2.5 (38) and gene expression was quantified with htseq-count (version 0.6.1p1) against Ensembl release 91. RNA-seq count matrices were subsequently prepared using the GenomicAlignments Bioconductor package (39). For differential expression analysis between Wt and *Hdac7^ko^* groups of one of the experimental stimulus, count matrices were subjected to the Bioconductor package DESeq2 as previously described (40). To correct for multiple testing issues, a Benjamini-Hochberg test was used to control false-discovery rates. For initial exploratory analyses and pathway analyses, differences in gene expression were considered significant if the p-adj was < 0.01.

For presenting an overview for each experimental condition, Volcano plots were generated that related the logarithmic fold change versus the adjusted p-value for each gene in *Hdac7^ko^* versus Wt cells. Differential gene expression signatures for each CD8 subpopulation was then analyzed using published mRNA expression signatures controlling memory formation and exhaustion in LCMV-specific CD8^+^ T cells (41) and canonical pathway analyses were performed using Ontologizer (42) for GO analysis and Fisher Exact Enrichment test using R (R Foundation). Heat maps show the logarithmic fold change. Adjusted p-values of differentially regulated genes of significantly enriched pathways were generated using the Prism 6 software package (GraphPad Software). For analyses comparing the expression of genes related to exhaustion, SOCE-, amino acid metabolism and apoptosis between *in vitro* activated Wt and *Hdac7^ko^* CD8^+^ T cells, genes were manually selected and considered to be differentially regulated if p-values were <0.05 and absolute log_2_-fold changes > 1.

**Figure 1:**
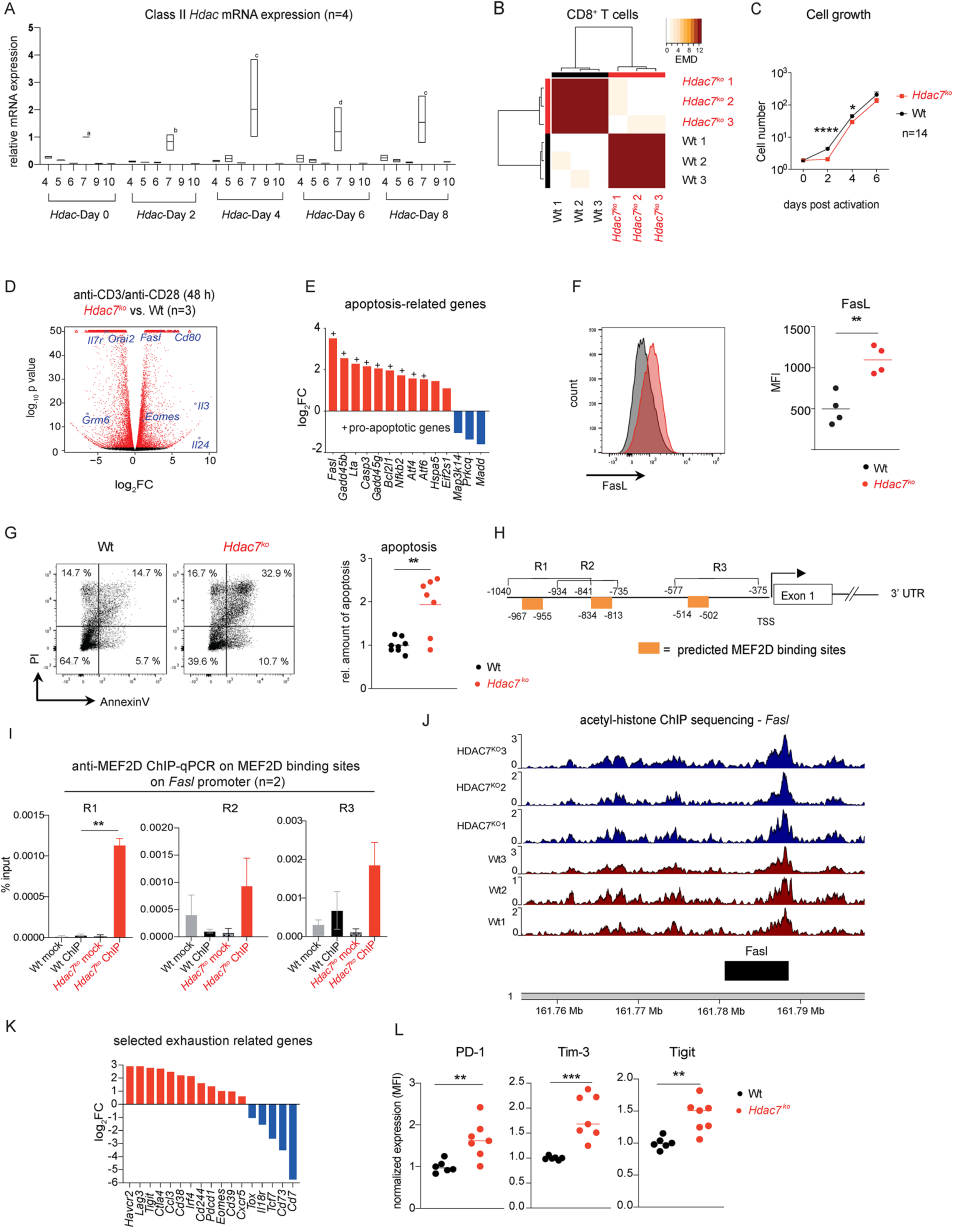
HDAC7 is crucial for the survival and the suppression of exhaustion markers in CD8^+^ T cells. **(A)** Box plots showing the mRNA expressions of Class II HDACs during *in vitro* CTL differentiation. Ct values were normalized to the Ct values of *36b4* and 2^->Ct^ was calculated. Expression of Class II HDACs are shown relative to the expression of *Hdac7* on Day 0 (n=4). p-value for HDAC7 vs. HDAC4–10 as follows: ^a^ p<0.05 vs all but HDAC4, ^b^ p<0.01 vs HDAC9 &10, ^c^ p<0.0005 vs all, ^d^ p<0.001 vs all. **(B)** Heatmap displaying the pairwise earth-mover’s distance (EMD) values of the cellular density distribution within CD8^+^ T cell population isolated from the spleens of Wt littermates or *Hdac7^fl/fl^-Cd4-Cre^+^(Hdac7^ko^)* mice after PMA/ionomycin stimulation over a 2D t-SNE space (n=3 for both groups). **(C)** Cell growth curve after *in vitro* activation with anti-CD3/CD28 antibodies of CD8^+^ T cells from Wt or *Hdac7^ko^* mice (n=14). **(D)** Volcano plots showing differentially regulated genes compared to Wt from comparative RNA-sequencing analysis of anti-CD3/CD28 antibody activated CD8^+^ T cells (n=3). **(E)** Fold-change expression of pro-apoptotic genes in *in vitro* activated *Hdac7^ko^* CD8^+^ T cells compared to the respective Wt CTL as assessed by RNA-sequencing (for all p<0.05, n=3). **(F)** Representative histograms showing FasL expression anti-CD3/CD28 stimulated Wt and *Hdac7^ko^* CD8^+^ T cells 2 days post activation (left) and MFI values of activated Wt and *Hdac7^ko^* CD8^+^ T cells (right; n=4, multiple t test). **(G)** Representative plots showing Annexin V and PI staining and the relative amount of apoptosis in Wt and *Hdac7^KO^* CTLs (right) 7 days post activation. Plots are representative of 3 independent experiments. Total frequencies of Annexin V^+^PI^+^ cells were normalized to the mean value of Wt CTLs (n=7-8, multiple t test). **(H)** Schematics of putative MEF2D binding sites at the murine *Fasl* promoter identified by *in silico* analysis. **(I)** Chromatin immunoprecipitation (ChIP)-qPCR analysis of MEF2D binding on the *Fasl* promoter on the MEF2D binding sites R1-R3. CD8^+^ T cells from *Hdac7^ko^* and Wt littermates were *in vitro* activated for 48 h with anti-CD3/CD28 antibodies and murine IL-2. ChIP was performed using anti-MEF2D antibody, followed by qPCR analysis. Plots show results of two independent experiments, in which 2–3 mice per group were pooled (multiple t test). **(J)** ChIP-seq was performed for Wt and *Hdac7^ko^* CD8^+^ T cells for acetylated histone 3 (K9/K14) and signal tracks for the *Fasl* locus are shown. Read density was calculated and visualized using the “karyoplotR”-package for each sample. (Wt: red, *Hdac7^ko^*: blue). **(K)** Fold change expression of CD8^+^ T cell exhaustion-related genes from comparative RNA-sequencing in *Hdac7^ko^* vs. Wt CD8^+^ T cells 48 h after *in vitro* activation with anti-CD3/CD28 antibodies. (For all p<0.05, n=3) and **(L)** Dot plots showing normalized protein expression of PD-1, Tim-3 and Tigit in Wt and *Hdac7^ko^* CD8^+^ T cells 48 h after *in vitro* activation with anti-CD3/CD28 antibodies. MFI values were normalized to the mean MFI of Wt. (3 independent experiments, n=6-7, multiple t-test) *p < 0.05; **p < 0.01, ***p < 0.001.

Re-analysis of single-cell sequencing data were performed as follows: count matrices were downloaded from GEO (GSE116390) und processed with Seurat (43), using IntegrateData to remove the systematic difference between the two conditions for clustering. Cluster identity was determined using marker genes from **Figure 1C** (44).

### CFSE dilution assay

CD8^+^ T cells were stained with 0.5 μM CFSE for 5 min at 37 °C followed by washing and re-suspension in complete RPMI 1640 medium. CFSE-labeled cells were *in vitro* activated as described above, and cells were analyzed by flow cytometry 3 days later. Cell division was analyzed using the FlowJo software (BD Biosciences).

### Intracellular Ca^+2^ measurement

*In vitro* differentiated CTLs were labeled with 2 µM Fura-2-AM (Invitrogen) for 20 min at room temperature, washed and re-suspended in RPMI 1640 medium. Subsequently, 4x10^5^ cells were cultured in poly-L-lysine (Sigma-Aldrich) coated 96-well plates and the Fura-2 emission ratio (340/380 nm) was measured using a FlexStation microplate reader (Molecular Devices). Baseline intracellular Ca2^+^ ([Ca^2+^]_i_) was acquired in 0 mM Ca^2+^ Ringer solution containing 155 mM NaCl, 4.5 mM KCl, 3 mM MgCl_2_, 10 mM D‐glucose, 5 mM Na-HEPES for 300 s as previously described (10). Subsequently, cells were stimulated with 1 µM thapsigargin (EMD Millipore, Billerica, MA). After 600 s, Ca^2+^ containing Ringer solution (2 mM CaCl_2_) was added to the cells (final [Ca^2+^]_o_ 1 mM). Cells were excited at 340 and 380  nm and fluorescence emission measured at 510 nm every 5 s. The Ca^2+^ influx rate (Δ*R*/Δ*t*) was calculated 20 s (*t*_20_) after re-addition of Ca^2+^ (*t*_0_) using the equation (*R*[*t*_20_] − *R*[*t*_0_])/(*t*_20_ − *t*_0_), with *R* being the Fura-2 emission ratio 340/380 nm. Graphs were plotted using Prism 6 software.

### Metabolic flux analyses

Cellular oxygen consumption rate (OCR) and extracellular acidification rate (ECAR) were measured using the Seahorse XFe96 Analyzer (Agilent) as previously described (45). Briefly, 300,000 cells/well in corresponding assay media (for OCR XF RPMI Assay medium supplemented with 1 mM pyruvate, 2 mM glutamine and 10 mM glucose and for ECAR 1 mM glutamine) were cultured into Seahorse XF96 Cell Culture Microplate, which was pre-coated with 10fold diluted poly-L-lysine (Sigma). Cells were centrifuged for complete attachment and incubated in a non-CO_2_ incubator at 37 C for 60 min. OCR was measured under basal conditions in response to 2 μM oligomycin (Sigma-Aldrich), 1 μM fluorocarbonyl cyanide phenylhydrazone (FCCP) (Sigma-Aldrich) and 0.5 μM rotenone + antimycin A (Sigma-Aldrich). Glutamine uptake was measured in response to stimulation with 4 mM glutamine. For glycolysis, ECAR was measured in response to 10 mM glucose, 1 μM oligomycin and 50 mM 2-deoxy-D-glucose (2-DG).

### Apoptosis assay

1x10^5^ *in vitro*-differentiated CTL were collected and washed with 1X Annexin V binding buffer (Thermo Fisher Scientific). Cells were stained with 1:100 Annexin V and incubated at room temperature for 5 min. Cells were washed with PBS containing 0.5% BSA and stained with propidium iodide and directly measured by flow cytometry. Flow cytometry data were analyzed by FlowJo software.

### Chromatin immunoprecipitation

CD8^+^ T cells were isolated from *Hdac7^fl/fl^Cd4-Cre* mice and wild type littermates and *in vitro* activated with anti-CD3 and anti-CD28 antibodies. 48 h after activation, 10x10^6^ cells were pooled and fixed using 1% formaldehyde for 5 min at room temperature by gentle shaking. Cells were then washed three times with ice-cold PBS and lysed in chromatin immunoprecipitation (ChIP)-lysis buffer (50 mM Tris pH 8.00, 10 mM EDTA, 1% SDS, complete protease (Roche) and phosphatase inhibitors (Sigma)) followed by chromatin sonication. Sonicated cell lysates were cleared by centrifugation and 2% of the samples were stored as input controls. Samples were diluted 4fold with ChIP-dilution buffer (16.7 mM Tris-HCl pH 8.0, 167 mM NaCl, 1.2 mM EDTA, 0.01% SDS, 1.1% Triton X-100). Subsequently, samples were pre-cleared with protein A agarose/salmon sperm DNA (Merck) for 1 hour at 4 °C on an overhead tumbler. Lysates were then incubated overnight with salmon sperm conjugated protein A agarose beads (Merck) and anti-MEF2D antibody or IgG at 4 °C on an overhead tumbler. Next day, agarose beads were precipitated by centrifugation at 100 g for 1 min followed by washing with high-salt (20 mM Tris-HCl pH 8.0, 500 mM NaCl, 2 mM EDTA, 0.1% SDS, 1% Triton X-100) and low-salt (20 mM Tris-HCl pH 8.0, 150 mM NaCl, 2 mM EDTA, 0.1% SDS, 1% Triton X-100) buffers. To elute DNA, precipitated protein A agarose bead-antibody complexes were incubated at 30 °C for 15 min in elution buffer (100 mM NaHCO_3_, 1% SDS) and subsequently treated with 1 µg RNaseA (Sigma-Aldrich) overnight at 65 °C. DNA was column-purified using a PCR purification kit (Macherey-Nagel) according to the manufacturer’s instructions for high-SDS containing samples.

### ChIP-qPCR

Input DNA and ChIP DNA samples were diluted in a 1:8 ratio and qPCR was performed using Sybr green reagent (Thermo Fisher Scientific) and the following primers: *Fasl*-Region 1 m*Fasl*_Chip1_Fo 5’-aggaacagcctgagattgc-3’ and m*Fasl*_Chip1_Re 5’-acctttttggcagactctacat-3’, *FasL*-Region-2 m*Fasl*_Chip2_Fo 5’-tgttttggcataggtgagag-3’ and m*Fasl*_Chip2_Re 5’-cacaccatttatgtctaataacc-3’, FasL-Region 3 m*Fasl*_Chip3_Fo 5’-gtaaatgttgaataatgttttagta-3’ and m*Fasl*_Chip3_Re 5’-ccctatccatcccacttc-3’. qPCR was analyzed using a StepOnePlus Real-Time PCR System. Fold enrichment of IP sample over mock (IgG) control was calculated with the % input method to calculate the relative enrichment of MEF2D on different sites of the *Fasl* promoter.

### ChIP-seq

Cells were harvested in complete growth medium and fixed by adding formaldehyde solution at a final concentration of 1% followed by incubation at room temperature for 5 min by gentle shaking. Fixed cells were washed with ice-cold PBS three times with centrifugation. Cell pellet was resuspended in cold chromatin immunoprecipitation (ChIP) lysis buffer including protease and phosphatase inhibitors and incubated at 4°C for 10 min. After cell lysis, chromatin sonication was performed. Lysed and sonicated samples were centrifuged at 14.000 g for 20 min to discard the debris. Two percent of the samples were stored as input controls and the rest was 4-fold diluted with ChIP dilution buffer supplemented with protease and phosphatase inhibitors. Sonicated samples were subsequently pre-cleared with salmon sperm blocked protein A agarose beads (Sigma-Aldrich) by rotating for 1 h at 4°C. Samples were centrifuged at 100 g for 2 min and supernatant was incubated with protein A beads and primary antibodies (anti-H3K9K14, Cell Signaling Technologies) control IgG. Next day, protein A agarose beads were washed with low- and high-salt buffers followed by DNA elution by incubation at 30 °C for 15 min. To reverse-crosslink ChIP DNA, elutes were incubated in ChIP elution buffer supplemented with RNase A. Next day, DNA was purified by column purification according to manufacturer’s instructions for high SDS containing samples (Macharey Nagel, Düren, Germany). The concentration of ChIP DNA was measured by Qubit device using Qubit High Sensitivity DSkit (Thermofisher Scientific) to selectively determine the concentration of double stranded DNA. The quality and the fragment size of DNA was assessed by agarose gel electrophoresis or Cell Free DNA Screen Tape Analysis (Agilent) according to manufacturer’s instructions. After library preparation, DNA samples were sequenced by Illumina sequencing according to manufacturer’s instructions.

### *In vitro* T cell exhaustion

CD8^+^ T cells were isolated from Wt mice and activated for 48 hours with anti-CD3 and anti-CD28 antibodies in the presence of recombinant IL-2. On day 2, cells were divided into two groups. While the experimental group was exposed to chronic antigen stimulation every two days to mimic repeated and chronic antigen stimulation, cells in the control group were grown in the presence of IL-2 only (46). The following qPCR primers were used for the detection of exhaustion marker gene expression: *Pdcd1*_Fo 5’-ACCCTGGTCATTCACTTGGG-3’ and *Pdcd1*_Re 5’-CATTTGCTCCCTCTGACACTG-3’, *Havcr2*_Fo 5’-GTAAGAATGCCTATCTGCCCTG-3^’^ and *Havcr2*_Re 5’-GCAACTCGTTGGTACACTGTGA-3’, *Tigit*_Fo 5’-CCACAGCAGGCACGATAGATA-3’ and *Tigit*_Re 5’-CATGCCACCCCAGGTCAAC-3’, *Lag3*_Fo 5’-CCTCGATGATTGCTAGTCCCT-3’ and *Lag3*_Re 5’-GTAGACAGGCACTCGGTTCTG-3’, *Ctla4*_Fo 5’-CATGGTGTCGCCAGCTTTC-3’ and *Ctla4*_Re 5’-GGTAATCTAGGAAGCCCACTGTA-3’. The following qPCR primers were used for the detection of amino acid transporter gene expression: Slc1a4-fo 5’-GGCATCGCTGTTGCTTACTTC-3’ and Slc1a4-re 5’-CGAGGAAAGAGTCCACTGTCT-3’, Slc1a5-fo 5’-CATCAACGACTCTGTTGTAGACC-3’ and Slc1a5-re 5’-CGCTGGATACAGGATTGCGG-3’, Slc7a1-fo 5’-CTTGGGCGCTGGTGTCTATG-3’ and Slc7a1-re 5’-CGTAGCTGTAGAGGTAGGCTG-3’, Slc7a5-fo 5’-CAGCTCCCTGAGTATGAAAGC-3’ and Slc7a5-re 5’-CCATTCCAGTAGACACCCCTTC-3’, Slc3a2-fo 5’-TGATGAATGCACCCTTGTACTTG-3’ and Slc3a2-re 5’-GCTCCCCAGTGAAAGTGGA-3’.

### Histopathology

Paraffin sections of 1 µm thickness were cut, dewaxed and subjected to a heat-induced epitope retrieval step. Endogenous peroxidase was blocked by hydrogen peroxide prior to incubation with anti-LAG-3 (polyclonal rabbit, Abcam #ab237720) followed by incubation with EnVision+ HRP-labelled polymer (Agilent). For visualization, the OPAL system according to manufacturer´s instructions (Akoya Biosciences) was used. Proteins were then inactivated and sections incubated with anti-PD-1 (clone D7D5W, Cell Signaling Technologies) followed by incubation with the EnVision+ polymer (Agilent) and the OPAL system (Akoya Biosciences). This staining cycle was repeated with anti-CTLA-4 (clone CAL49, Abcam), anti-CD8 (clone D8A8Y, Cell Signaling Technologies), and anti-Tim3 (clone D3M9R, Cell Signaling Technologies). The following OPAL dyes were used for visualization: OPAL-570 for LAG-3, OPAL-620 for PD-1, OPAL-540 for CTLA-4, OPAL-520 for CD8, and OPAL650 for Tim-3. All antibodies including clone numbers and their dilutions are listed in **Supplementary Table 5**. Nuclei were stained using 4′,6-diamidine-2′-phenylindole dihydrochloride (DAPI; Sigma) and slides were coverslipped in Fluoromount G (Southern Biotech). Multispectral images were acquired using a Vectra^®^ 3 imaging system (Akoya Biosciences). The inForm software (version 2.4.8) was used for spectral unmixing and cell segmentation as well as cell phenotyping. The cell phenotypes were quantified using RStudio (version 1.3.1073).

### Tumor allografts

EG.7-Ova cells were purchased from ATCC. Gerald Willimsky (Charité – Universitätsmedizin Berlin) kindly provided B16-Ova cells. EG.7-Ova and B16-Ova cells were cultured in RPMI 1640 and DMEM medium respectively, supplemented with 10% fetal calf serum, penicillin streptomycin solution and 500 µg/ml G418 (Sigma-Aldrich). 1x10^5^ B16-Ova and 1x10^6^ EG.7-Ova cells were intradermally (i.d.) injected into the flank of mice. Tumor size was assessed *in situ* by using a caliper. Tumor weights were measured after tumor excision.

### Adoptive transfer of cytotoxic T cells

Ova-specific cytotoxic T cells (CTLs) were generated by isolating splenocytes of *Hdac7^fl/fl^Cd4-Cre-OT1-Thy1.1* mice or Wt littermates followed by magnetic enrichment of CD8^+^ T cells using a negative murine CD8^+^ T cell isolation kit (Stemcell). CD8^+^ T cells were *in vitro* activated using 2 µg/ml anti-CD3 and anti-CD28 antibody and 20 ng/ml murine IL-2 (Peprotech) followed by an *in vitro* expansion in the presence of 20 ng/ml murine IL-2. On day 7, CTLs were either used for adoptive CTL-transfer experiments or for *in vitro* assays. To investigate the *in vivo* persistence of Wt-OT1-Thy1.1^+^ and *Hdac7^ko^*-OT1-Thy1.1^+^ CTLs, 7x10^6^ *in vitro* differentiated CTLs were simultaneously injected with 1x10^6^ EG.7-Ova cells into Thy1.2^+^ *Hdac7^ko^* mice and the frequencies of transferred CTLs in spleens and TDLN were analyzed 7 days post transfer by flow cytometry. For rescue experiments in *Hdac7^ko^* mice, EG.7-Ova tumors were established in Thy1.2 Wt mice by i.d. injection of 1x10^6^ EG.7-Ova cells. Seven days later, mice with established tumors received i.v. injections with 8x10^6^ Ova-specific CTLs and tumor growth was subsequently monitored. Seven days after adoptive transfer, recipient mice were sacrificed and the frequencies as well as PD-1 and Tim-3 expression of Thy1.1^+^ cells were analyzed in spleens, tumor draining lymph nodes (TDLN) and tumors by using flow cytometry. For homing experiments, B16-Ova tumors were established in Wt mice with i.d. injection of 5x10^5^ cells and 10 days later mice with established B16-Ova tumors were i.v. injected with 7x10^6^ CFSE-labeled Ova-specific *Hdac7^ko^* or Wt CTLs. Recipient mice were euthanized 90 min after i.v. transfer and TDLN and tumor infiltrating lymphocytes were harvested. Subsequently, the percentage of CFSE^high^ CD8^+^ T cells was analyzed by flow cytometry.

### LCMV infection models

The lymphocytic choriomeningitis virus Armstrong strain (clone 53b) (LCMV_Arm_) and the LCMV clone 13 (LCMV_cl13_), were propagated and stored as previously described (47). Shortly, the LCMV_Arm_ was propagated on BHK21 cells and the stock concentration was determined using a modified plaque assay on Vero cells (48). For primary infections, mice were infected i.p. with 2×10^5^ pfu LCMV_Arm_. For secondary infections with LCMV_cl13_), mice were injected intravenous (i.v) with 2×10^6^ pfu LCMV_cl13–_60 days after prior infection with LCMV_Arm_.

### Cell preparation, *in vitro* stimulation and flow cytometric analyses of splenocytes from LCMV-infected mice

Single cell suspensions were obtained by passing whole spleens through 70 µm filters followed by the removal of erythrocytes using ACK-Lysis buffer (Gibco). For absolute cell counts a defined volume of cell suspension was stained with DAPI, anti-CD90, -CD8 and -CD4 antibodies and analyzed using a MACS Quant flow cytometer (Miltenyi Biotec). For the assessment of cytokines, isolated cells were stimulated for 6 h at 37 °C with the cognate LCMV Gp33 (GP_33-41_) peptide (Anaspec, 1 µg/ml, KAVYNFATM), Gp267 (GP_276-286_; Anaspec, 1 µg/ml, SGVENPGGYCL) or Gp61 (GP_61-80_; Anaspec, 1 µg/ml, GLNGPDIYKGVYQFKSVEFD) in the presence of 0.01 mg/ml brefeldin A (Sigma-Aldrich). For the detection of LCMV-specific CD8^+^ T cells, splenocytes were incubated with MHC I-streptamer H-2D^b^ LCMV (IBA) and conjugated with Strep-Tactin PE (IBA) according to the manufacturer’s protocol. Dead cells were excluded by the use of Pacific Orange dye (Invitrogen). Intracellular staining was performed for 30 min at 4 °C after fixation and permeabilization with FACS-Lysing and FACS-Perm2 Solution (BD Bioscience) according to the manufacturer’s protocol. All information on antibody dilutions, fluorochromes and catalogue numbers are provided in **Supplementary Table 3**. All fluorochrome-labeled samples were either measured with BD FACSCanto II or BD LSRII SORP (BD Bioscience) and analyzed using the FlowJo v10.1 software package (BD Bioscience) following the guidelines for the use of flow cytometry and cell sorting in immunological studies (49).

### Analysis of viral titers

Detection of viral RNA was performed as described before (47). Shortly, viral RNA was isolated from serum of LCMV infected mice using the Nucleospin RNA virus kit (Macherey-Nagel) according to manufacturer’s protocol. For viral RNA quantification, a 401-nt-long dsDNA strand was amplified with primers NP1808f and NP2206r-T7 overhang from a plasmid template kindly provided by Daniel Pinschewer (Department of Biomedicine, University of Basel) containing the NP-ORF of the LCMV_Arm_. After gel extraction (Nucleospin Gel and PCR clean-up kit, Macherey-Nagel), the RNA strand was generated by T7 *in vitro* transcription, subsequent DNA digestion, and quantified with NanoDrop2000 (Thermo Fisher Scientific). Standard serial dilution and samples were analyzed by StepOnePlus Real-Time PCR System (Applied Biosystems) as previously described.

### Immunoblotting

For immunoblotting acetyl-Histone 3 Lysine9/Lysine14 (all Cell Signaling Technology) and β-actin (Sigma-Aldrich) (**Supplementary Table 4**), CTLs were lysed in radioimmunoprecipitation assay buffer (RIPA) including complete protease inhibitors (Roche) and phosphatase inhibitor cocktail (Sigma). 30 µg of whole cell lysates were run in 8% SDS-PAGE gels. For acetylated-histone 3, 15% SDS-PAGE was used. Proteins were blotted at 250 mA for 1–2 h in a wet tank to PVDF membrane. Membranes were blocked with 5% BSA-TBST solution and incubated overnight with primary antibodies at 4 °C on a rotator. All primary antibodies were diluted 1:1000 in 5% BSA-TBST solution except for anti-β-actin which was 1:2000. On the consecutive day, membranes were washed with 1X TBS-T solution and incubated with 1:2000 diluted HRP-linked secondary antibodies (Dako) in 2.5% BSA-TBST solution for 1 h at room temperature. The detection was performed on luminescent image analyzer LAS-4000 mini (Fuji Film) using the WB detection ECL reagent (GE Healthcare). Densiometric analysis was performed using the ImageJ (1.48V) software.

### Glutamine consumption assay

Wt and *Hdac7^ko^* CD8^+^ T cells activated with α-CD3 and α-CD28 antibodies for 7 days were collected and carefully counted multiple times to ensure accurate cell density before being resuspended in glutamine-free medium (Seahorse RPMI medium, Agilent) at a density of 10 x 10^6^ cells/mL. Two million cells were seeded into 96-well plate in triplicate and mixed with an equal volume of 0.5 μCi L-2,3,4 [^3^H] glutamine (Perkin Elmer)-containing tracer buffer by pipetting. Before the incubation with radioactive glutamine, cells were incubated in the glutamine-free medium for 2 hours. During a 4-minute incubation period at room temperature, the content of each well was transferred to 1.5 mL Eppendorf tubes preloaded with 500 μL oil mixture containing a 1:1 ratio of Poly(dimethylsiloxane-co-methylphenylsiloxane) (Sigma-Aldrich) and Diisononyl phthalate (Sigma-Aldrich), carefully layering the mixture on top. The uptake of glutamine was terminated by pelleting down the cells at 8500 rcf at 4°C, followed by the removal of the aqueous supernatant. The remaining radioactivity on the tube walls was rinsed with 500 μL distilled water. After discarding the final rinse and the oil mixture, cells were lysed and resuspended in 200 μL 1 M NaOH. The lysate was then transferred into scintillation vials and brought to a final volume of 3 mL with scintillant (Optiphase HiSafe3, Perkin Elmer). Finally, β-radioactivity of the samples was measured in counts per minute (cpm) using a scintillation counter.

### Mitochondrial dye staining

For each sample, 0.3 x 10^6^ cells were seeded into a 96-well plate, washed once with pre-warmed serum-free medium, and incubated with 250 nM MitoTracker™ Red CMXRos (Thermo/Invitrogen) in serum-free medium at 37°C for 30 minutes and subsequently stained with 50µL Zombie-Aqua (Biolegend) and analyzed by flow cytometry.

### FasL neutralization

Cells were activated for 48 hours in accordance with the *in vitro* differentiation protocol as described. For the *in vitro* chronic antigen stimulation model, 1x 10^6^ activated cells were re-activated in 2 mL medium within 12-well plates coated with 1 μg/mL anti-CD3 and anti-CD28 antibodies, without addition of recombinant IL-2. FasL neutralization was performed by adding 20 µg/mL anti-mouse FasL antibody (clone MFL3, BioCell, BE0319) to the culture medium, either during the initial activation or at the time of re-stimulation.

## Results

### Deletion of *Hdac7* in T cells results in a pre-activated phenotype of CD8^+^ T cells

The epigenetic regulators controlling the homeostasis of adult CD8^+^ T cells are incompletely understood and the specific role of class II HDACs in the differentiation and function of effector and memory CD8^+^ T cells as well as their contribution to the regulation of cellular exhaustion remain elusive. To delineate which class II HDAC members are expressed in peripheral CD8^+^ T cells, we first determined the mRNA expression levels of class II HDAC members during CTL differentiation of wild type (Wt) CD8^+^ T cells *in vitro*. We observed that *Hdac7* was the most highly transcribed class II member at all investigated time points (**Figure 1A**). To elucidate the role of HDAC7 in the differentiation and function of T cells, we subsequently generated conditional *Hdac7* knockout (*Hdac7^ko^*) mice by crossing *Hdac7^fl/fl^* and *Cd4-Cre* mice, which resulted in a deletion of *Hdac7* both in CD4^+^ and CD8^+^ T cells (**Supplementary Figures 1A**, **4A, C**). We next compared the splenic T cell composition of *Hdac7^ko^* or Wt mice by mass cytometry, using two independent panels of 31 and 35 lineage and functional markers (**Supplementary Tables 1**, **2**) and by subsequently applying the t-SNE algorithm on pre-gated CD3^+^ T cells. Thereby, we identified a lack of naïve CD62L^+^CD44^-^CD8^+^ T cells in *Hdac7^ko^* mice (**Supplementary Figure 1B**). These phenotypical differences became even more evident, when the clustering of earth mover’s distance (EMD) values of all mass cytometry markers after *ex vivo* stimulation with PMA/ionomycin was analyzed. Here, CD8^+^ T cells of *Hdac7^ko^* mice varied significantly from those of Wt mice (**Figure 1B**), whereas CD4^+^ T cells displayed a closer similarity between both groups (**Supplementary Figure 1C**), suggesting that HDAC7-deficiency is mainly affecting peripheral CD8^+^ T cells but not CD4^+^ T cells. Accordingly, Wt and *Hdac7^ko^* CD4^+^ T cells shared the same colitogenic potential in an adoptive T cell transfer model of colitis, as no significant differences in disease severity could be detected between recipient *Rag2^-/^*^-^ mice that had either received naïve Wt or *Hdac7^ko^* CD4^+^ T cells (**Supplementary Figures 1E–H**) although in the steady state, minor variations in the ability of HDAC7-deficient CD4^+^ T cells to produce IL-17A and TNFα could be observed (**Supplementary Figure 1D**).

### Increased apoptosis in HDAC7-deficient CD8^+^ T cells

To better characterize the impact of HDAC7-deficiency on the homeostasis of peripheral CD8^+^ T cells, we next compared the expansion and the transcriptome of *Hdac7^ko^* and Wt CD8^+^ T cells after *in vitro* activation with anti-CD3/CD28 antibodies. Interestingly, the cell number of *Hdac7^ko^* CD8^+^ T cells was significantly reduced 2–4 days after initial stimulation (**Figure 1C**) despite a comparable proliferative capacity of *Hdac7*-deficient and Wt CD8^+^ T cells (**Supplementary Figures 2A–C**). RNA sequencing of *in vitro* activated naive CD8^+^ T cells from either Wt or *Hdac7^ko^* mice revealed that several pro-apoptotic genes were upregulated in *Hdac7^ko^* CD8^+^ T cells compared to Wt cells (**Figures 1D, H**) of which FasL expression could also be validated on the protein level (**Figure 1F**), ultimately leading to an increased rate of apoptosis and impaired survival of *Hdac7^ko^* CD8^+^ T cells (**Figure 1G**). Since HDAC7 is known to interact with and repress MEF2 family transcription factors and MEF2D is a known inducer of pro-apoptotic genes in T cells (32), we hypothesized that the observed increased expression of FasL in *Hdac7^ko^* CD8^+^ T cells might be due to an enhanced binding of MEF2D to the *Fasl* promoter. Likewise, we observed that MEF2D is significantly enriched in *Hdac7^ko^* CD8^+^ T cells on three predicted binding sites (R1-R3) within the *Fasl* promoter (**Figures 1H, I**) thereby inducing the expression of *Fasl*. In parallel, applying acetyl-histone chromatin immunoprecipitation with a subsequent sequencing (ChIP-Seq), no differences in chromatin-acetylation in *Hdac7*^ko^ cells compared to Wt could be detected, neither for the FasL gene nor the respective promoter region (**Figure 1J**).

Using immunoblotting and chromatin immunoprecipitation followed by sequencing (ChIP-seq) we next examined to which extent HDAC7 controls transcription in CD8^+^ T cells via its function as histone-deacetylase. *Hdac7^ko^* T cells displayed global (**Supplementary Figures 3A, B**, **4A, B**) and locus-specific changes in H3K9ac/H3K14ac, including at metabolism/apoptosis-related genes such as *Acox3*, *Foxo4*, and *Bcl2l13* (**Supplementary Figures 3C, D**), indicating potential regulatory functions of HDAC7 on the epigenetic level. However, we could not observe statistically significant differences in histone acetylation at the *Fasl* locus between Wt and *Hdac7^ko^* CD8^+^ T cells, indicating that HDAC7 primarily regulates *Fasl* expression through a MEF2D-dependent mechanism rather than through its deacetylase activity.

### HDAC7-dependent control of calcium homeostasis and metabolism

RNA sequencing analyses of *in vitro* synchronized CD8^+^ T cells from either Wt or *Hdac7^ko^* mice furthermore revealed the transcriptional deregulation of several key components of SOCE-signaling in HDAC7-deficient CD8^+^ T cells including the significant downregulation of the calcium channel protein *Orai2*, the adaptor molecule *Stim1*, the sarco/endoplasmic reticulum Ca^2+^-ATPase (SERCA) pump *Atp2a2* as well as inositol-3-phosphate receptors (*Itpr*)*2* and *Itpr3*. These findings indicate that HDAC7 is required for proper Ca^2+^ homeostasis in CD8^+^ T cells as HDAC7-deficient CD8^+^ T cells also display a significantly decreased calcium influx after stimulation with thapsigargin when compared to Wt cells (**Supplementary Figures 2D, E**).

Furthermore, ChIP-Seq analysis (**Supplementary Figures 8A, B**) revealed differentially acetylated histones at loci including *Acox3*, *Foxo4* and *Bcl2l13*, genes associated with metabolic regulation as β-oxidation, oxidative stress or mitochondrial-mediated apoptosis and which are found upregulated in activated *Hdac7^ko^* CD8^+^ T cells compared to Wt (**Supplementary Figures 8C, D**).

**Figure 2:**
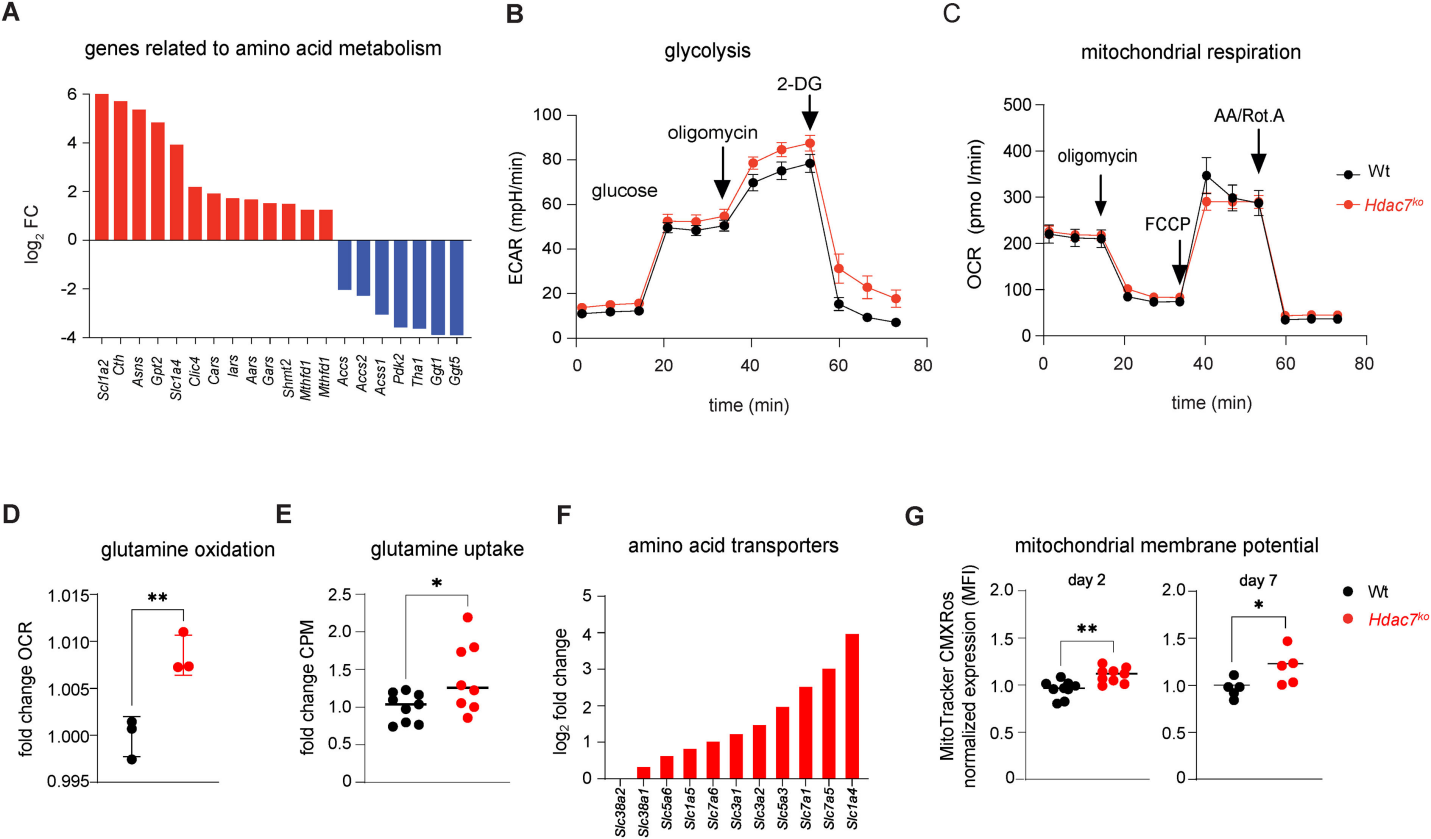
**(A)** Naïve Wt and *Hdac7^ko^* CD8^+^T cells were activated *in vitro* with anti-CD3 and anti-CD28 antibodies for 48 hours, and RNA samples were subjected to bulk RNA sequencing. Fold change expression of amino acid metabolism related genes in *Hdac7^ko^* CD8^+^ T cells compared to Wt cells from RNA-sequencing (for all p<0.05, n=3 per group). (B/C) Metabolic flux measurements of Wt and *Hdac7ko* CTLs 7 days post activation. **(B)** Glycolysis levels as assessed by extracellular acidification rate (ECAR) values measured in response to 10 mM glucose, 1 μM oligomycin and 50 mM 2-DG treatment (n=3, biologically independent samples). **(C)** Mitochondrial respiration levels assessed by oxygen consumption rate (OCR) values measured in response to 2 μM oligomycin, 1 µM FCCP and 0.5 µM RotA/AA treatment (n=3, biologically independent samples). **(D)** Mitochondrial respiration levels assessed by oxygen consumption rate (OCR) values. Glutamine oxidation assessed by OCR levels measured in response to 4 mM glutamine treatment. (n=3, biologically independent samples, unpaired t-test) **p<0.01. **(E)** H^3^-labeled glutamine consumption assay performed in WT and *Hdac7^ko^* CD8^+^ T cells activated with anti-CD3 and anti-CD28 antibodies for 7 days. Glutamine uptake was quantified by scintillation counting of the cellular lysate. Fold change was generated by normalizing H^3^ CPM values of all data points to the average H^3^ CPM value of WT group of each experimental batch. Three independent experiments, n=8–9 biological replicates each, unpaired t test, *p<0.05. **(F)** As in **(A)**, amino acid transporter expression is shown as log2 fold change, Wt vs. *Hdac7^ko^*. **(G)** Flow cytometry analysis of mitochondrial membrane potential were performed in WT and *Hdac7^ko^* CD8^+^ T cells activated with anti-CD3 and anti-CD28 antibodies for 2 or 7 days. Fold change was generated by normalizing MFI values of all data points to the average value of WT group of each experimental batch. (three to four independent experiments, n=5-8, unpaired t test= *p<0.05, **p<0.01.

Consistent with our RNA-seq data showing the upregulation of amino acid metabolism-related genes in *Hdac7^ko^* CD8^+^ T cells, (**Figure 2A**, **Supplementary Figures 2F, G**), metabolic flux analyses revealed that while HDAC7-deficiency does not affect glycolysis and mitochondrial metabolism in CTLs, it results in increased glutamine oxidation in HDAC7-deficient CTLs when compared to Wt CTLs (**Figures 2B–D**). Deeper analyses demonstrated a higher expression of glutamine receptors as well as an increased glutamine uptake in *Hdac7^ko^* CD8^+^ T cells, paralleled by an enhanced mitochondrial membrane potential (**Figures 2E, G**; **Supplementary Figure 3**), which does not indicate a uniform increase in mitochondrial fitness but rather point to an altered metabolic state associated with activation.

In summary, our data demonstrate that HDAC7 plays crucial roles for the survival, calcium homeostasis as well as the metabolic fitness of peripheral adult CD8^+^ T cells.

### HDAC7 is needed to suppress the upregulation of exhaustion markers during repeated antigen stimulation

Interestingly, we also observed a significant transcriptional upregulation of various well known CD8^+^ T cell exhaustion markers including *Havcr2*, *Ctla4, Pdcd1*, *Lag3* and *Tigit* by RNA sequencing in CD8^+^ T cells from *Hdac7^ko^* mice 48 h after *ex vivo* stimulation with anti-CD3/CD28 antibodies, suggesting a crucial role for HDAC7 for the suppression of exhaustion markers in these cells (**Figures 1K, L**).

**Figure 3:**
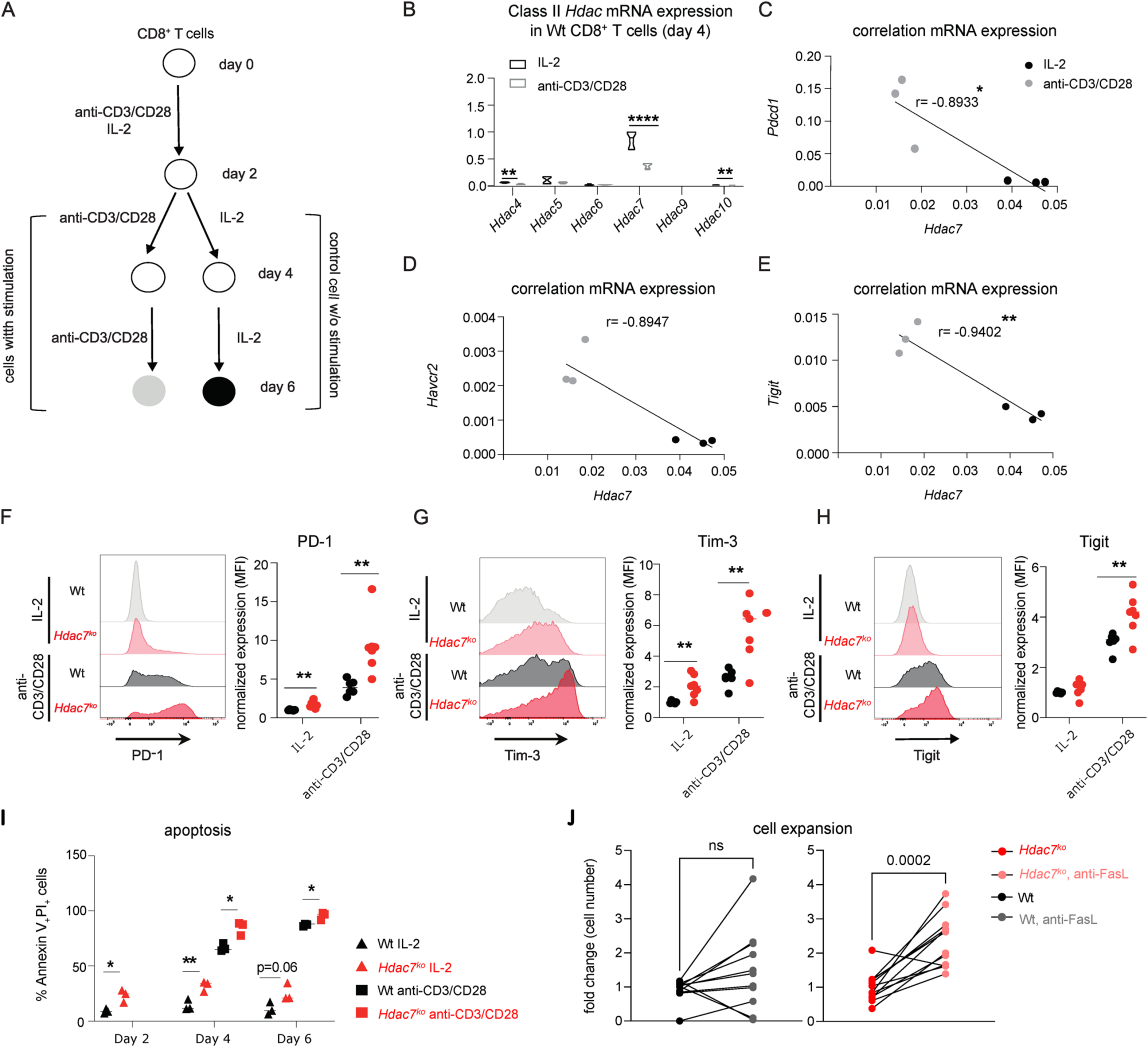
HDAC7 deletion in CD8^+^ T cells results in the upregulation of exhaustion markers and metabolic changes during repeated antigen stimulation. **(A)** Experimental setup for *in vitro* chronic T cell stimulation model. Wt CD8^+^ T cells were *in vitro* activated with anti-CD3/CD28 antibodies for 48 h and cultured in the presence of recombinant IL-2 only or restimulated with anti-CD3/CD28 antibodies for an additional 48 h. **(B)** Relative mRNA expression of class II HDACs upon restimulation with anti-CD3/CD28 on day 4 relative to control cells grown in the presence of IL-2 only without repeated stimulation. Ct values were normalized to the Ct values of *36b4* and 2^-Ct^; was calculated (n=4, multiple t test). **(C–E)** Representative graphs showing the correlation between mRNA expressions of *Hdac7* and *Pdcd1*, *Havcr2* and *Tigit*. Pearson correlation analysis with 95% confidence interval (n=6). **(F–H)** Representative primary FACS plots and dot plots showing the expression of **(F)** PD-1, **(G)** Tim-3 and **(H)** TIGIT in Wt or *Hdac7^ko^* CD8^+^ T cells with or without restimulation *in vitro* (multiple t test, 3 independent experiments, n=6-7). **(I)** Apoptosis measured by Annexin V, 7AAD staining via flow cytometry analysis with or without anti-FasL antibody supplement. Fold change was generated by normalizing the frequency of AnnexinV^+^7AAD^+^ of all data points to the average value of WT group of each experimental batch. (four independent experiments, n=10-12, unpaired t test). **(J)** Cell count generated by automatic cell counter for WT and Hdac7^ko^ CD8^+^ T cells with or without anti-FasL antibody supplement. Fold change was generated by normalizing all data points to the average value of WT group of each experimental batch. (four independent experiments, n=10-12, unpaired t test). *p<0.05, **p < 0.01, ***p < 0.001, ****p < 0.0001.

To further investigate if HDAC7 contributes to the suppression of T cell exhaustion in CD8^+^ T cells, we next examined the correlation of *Hdac7* expression with the expression of exhaustion markers in Wt CD8^+^ T cells in an *in vitro* model of chronic T cell stimulation (46). Thus, we isolated CD8^+^ T cells from Wt mice and activated them for 48 hours with anti-CD3/CD28 antibodies in the presence of recombinant IL-2. On day 2, cells were divided into two groups: While the experimental group was exposed to repeated stimulation with anti-CD3/CD28 every other day to mimic chronic antigen stimulation, cells in the control group were grown in the presence of IL-2 only (46) (**Figure 3A**). We then first determined whether the expression of *Hdac7* and the other class II *Hdac* genes were affected by repeated stimulation in CD8^+^ T cells. Interestingly, the expression of *Hdac7* significantly decreased upon repeated re-stimulation of cells (**Figure 3B**). Next, we analyzed how *Hdac7* expression was correlated with the expression of exhaustion marker genes. Significant negative correlations were observed between *Hdac7* expression and *Pdcd1*, *Havcr2* and *Tigit* (**Figures 3C–E**), which were upregulated in Wt CD8^+^ T cells upon repeated anti-CD3/CD28 stimulation (**Supplementary Figure 4A**). In accordance, we detected a significantly higher protein expressions of PD-1, Tim-3 and Tigit in *Hdac7^ko^* CD8^+^ T cells compared to Wt when we applied our *in vitro* model of repeated T cell stimulation on *Hdac7^ko^* CD8^+^ T cells (**Figures 3F–H**). These results suggest that HDAC7 acts as a repressor of CD8^+^ T cell exhaustion-markers under chronic and repeated antigen stimulations.

Remarkably, this increase in the expression of cellular exhaustion markers was also associated with elevated levels of apoptosis in CD8^+^ T cells lacking HDAC7 demonstrating that *Hdac7^ko^* CD8^+^ T cells are more prone to develop apoptosis during chronic stimulation, which is also a hallmark of exhausted CD8^+^ T cells (41) (**Figure 3I**). In our *in vitro* model of repeated T cell stimulation, apoptosis induction in *Hdac7^ko^* CD8^+^ T cells could be rescued by neutralizing anti-FasL antibody (**Figure 3J**; **Supplementary Figure 4B**). Remarkably, differences in FasL expression were strongest at early timepoints after stimulation (day 2) and equalized at day 7 (**Supplementary Figure 4C**). Taken together these data suggest that HDAC7 is not only required for the survival of CD8^+^ T cells during acute and continuous stimulation, but that it is also crucial to prevent cellular exhaustion of CD8^+^ T cells.

### Impaired anti-tumor immunity in *Hdac7^ko^* mice

**Figure 4:**
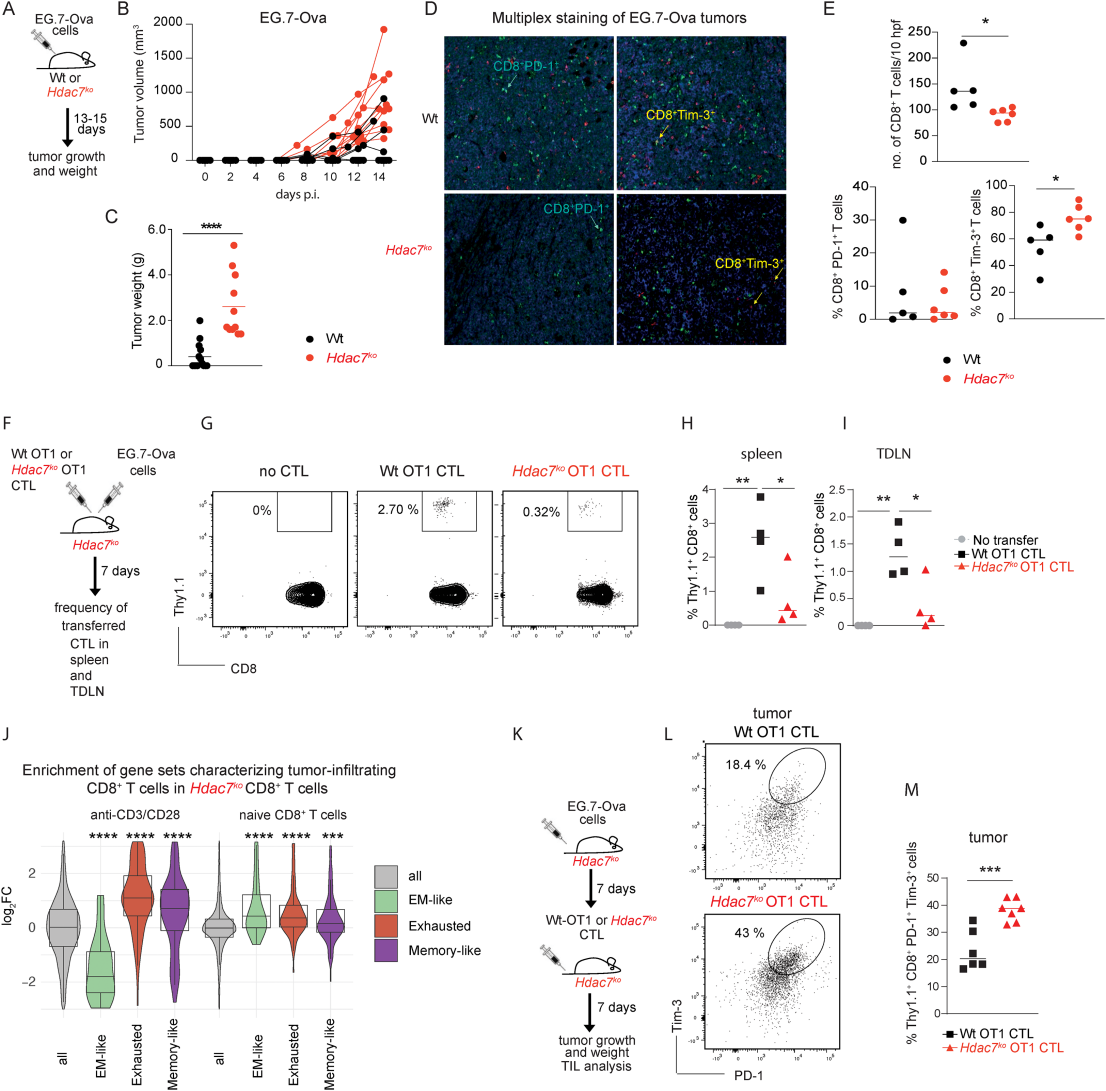
HDAC7 controls infiltration, *in vivo* persistence and the suppression of cellular exhaustion of CD8^+^ T cells in the tumors. **(A)** Wt and *Hdac7^fl/fl^Cd4-Cre (Hdac7^ko^)* mice were intradermally (i.d.) injected with 1x10^6^ EG.7-Ova cells. Tumor growth was followed for 14 days. **(B)** Tumor growth in Wt and *Hdac7^ko^* mice shown as increase in tumor volumes (n=12–14 mice per group). **(C)** Tumor weight in Wt and *Hdac7^ko^* mice on day 14 after tumor inoculation (n=12–14 animals per group, multiple t test). **(D)** Representative images from multiplex staining of EG.7-Ova tumor tissues in Wt (above) and *Hdac7^ko^* (below) mice (CD8=green, PD-1=blue, Tim-3=red). **(E)** Number of tumor-infiltrating CD8^+^ T cells (upper panel) and the frequencies of CD8^+^PD-1^+^ and CD8^+^Tim-3^+^ cells (lower panels) per 5 high power fields (hpf) in EG.7-Ova tumors in Wt and *Hdac7^ko^* mice 14 days p.i. (n=5–6 mice per group, multiple t test). **(F)** *Hdac7^ko^* mice were injected with EG.7-Ova cells. Wt-OT1 or *Hdac7^ko^*-OT1 CTLs were transferred on the same day. The frequencies of transferred cells were analyzed after 7 days in spleens and tumor-draining lymph nodes (TDLN). **(G)** Representative flow cytometry plots showing the splenic frequencies of Thy1.1^+^ Wt OT1 or Thy1.1^+^*Hdac7*^ko^-OT1 CTLs. **(H, I)** Dot plots of frequencies in spleens and TLDN of Wt OT1 and *Hdac7*^ko^-OT1 CTLs 7 days after i.v. transfer (n=4 mice per group, multiple t test). **(J)** *In silico* enrichment analysis of published gene sets^33^ obtained from tumor-infiltrating CD8^+^ T cells in anti-CD3/CD28 activated *Hdac7^ko^* CD8^+^ T cells compared to the respective Wt (Wilcoxon test). Gene sets were compared to all enriched genes (all). **(K)** *Hdac7^ko^* mice were injected (i.d.) with 1x10^6^ EG.7-Ova cells. Seven days after tumor inoculation, recipient mice were injected with 7x10^6^ *in vitro* differentiated Wt OT1 or *Hdac7^ko^* OT1 CTLs. Tumor growth was followed for 7 days post-CTL transfer. **(L)** Representative FACS plots showing PD1^+^Tim3^+^Thy1.1^+^ CTLs in EG.7-Ova tumors in transferred mice. **(M)** Dot plot showing the frequencies of Thy1.1^+^PD1^+^Tim-3^+^CD8^+^ Wt OT1 and *Hdac7^ko^* OT1 cells within EG.7-Ova tumors (n=6-7, multiple t test). *p< 0.05; **p < 0.01, ***p< 0.001, ****p<0.0001.

To address if HDAC7 also controls T cell survival and exhaustion during chronic and repetitive antigen-exposure *in vivo*, we next explored how the deletion of *Hdac7* affects anti-tumor immune responses of CD8^+^ T cells in mice. Thus, we challenged *Hdac7^ko^* mice or Wt littermates with syngeneic murine EG.7-Ova lymphoma cells (**Figure 4A**), which resulted in increased tumor volume and tumor weight in *Hdac7*^ko^ mice (**Figures 4B, C**). These results were confirmed in *Hdac7^fl/fl^-E8I-Cre* mice (**Supplementary Figures 5A–C**), in which HDAC7 is exclusively deleted in CD8^+^ T cells, supporting a CD8^+^ T cell intrinsic function of HDAC7 in proper anti-tumor immune responses. As shown in **Figures 4D, E**, significantly fewer tumor infiltrating CD8^+^ T cells could be detected in tumors of *Hdac7^ko^* mice, which was paralleled by an increased frequency of CD8^+^Tim-3^+^ cells suggesting that HDAC7 might be required for either the expansion, survival, exhaustion and/or the homing of tumor-specific CD8^+^ T cells.

To exclude an impairment in the homing capacity of HDAC7-deficient CD8^+^ T cells, we transferred either *in vitro* expanded Wt *Thy1.1^+^Ot-1^+^* (Wt-OT1) CTLs or *Hdac7^ko^ Thy1.1^+^Ot-1^+^* (*Hdac7^ko^*-OT1) CTLs into recipient Wt mice with established tumors and compared the frequency of tumor-infiltrating CD8^+^ T cells 90 min after intravenous (i.v.) transfer (**Supplementary Figure 5D**). As shown in **Supplementary Figure 5E**, no statistically significant difference in the homing capacity to either tumors or tumor draining lymph nodes (TDLN) could be detected in mice receiving *Hdac7^ko^* or Wt CTL. To clarify whether HDAC7 is required to maintain the survival of CD8^+^ T cells *in vivo*, we subsequently injected recipient *Hdac7^ko^* mice with the same number of Thy1.1^+^ Wt-OT1 or *Hdac7^ko^*-OT1 CTLs and simultaneously challenged these mice with EG.7-Ova lymphoma cells (**Figure 4F**). Indeed, we observed a reduced frequency of transferred Thy1.1^+^ *Hdac7^ko^* CTLs in spleens as well as TDLN, 7 days after adoptive transfer, implying that HDAC7 is needed for the *in vivo* persistence of transferred CTLs (**Figures 4G–I**). In accordance with our previous experiments, we also noted a significant enrichment of gene-signatures in the transcriptome of HDAC7-deficient CD8^+^ T cells that have been associated with the exhaustion of tumor-infiltrating CD8^+^ T cells (44) (gene-signatures were derived from the re-analysis of published single-cell RNA sequencing data of melanoma infiltrating CD8^+^ T cells, **Figure 4J** and **Supplementary Figures 5F, G**). To confirm that the lack of HDAC7 in tumor infiltrating CTLs indeed results in an exhausted phenotype, CD8^+^ T cells from Wt OT1 or *Hdac7^ko^* OT1 mice were *in vitro* differentiated into CTLs and subsequently i.v. injected into recipient EG.7-Ova tumor bearing *Hdac7^ko^* mice (**Figure 4K**), which significantly delayed tumor growth in both experimental groups when compared to EG7-Ova bearing mice not receiving CTLs (**Supplementary Figures 5H, I**). However, the analysis of EG.7-Ova infiltrating CD8^+^ T cells revealed that tumor-infiltrating *Hdac7^ko^* OT1 CD8^+^ T cells display a higher abundance of PD1^+^Tim3^+^ cells, implying that *Hdac7*-deficient CD8^+^ T cells are more prone to develop cellular exhaustion under chronic antigen stimulation within tumors (**Figures 4L, M**), which could not be observed in transferred *Hdac7^ko^* CD8^+^ T cells in spleens or TDLN (**Supplementary Figures 5J, K**).

### Dysfunctional memory recall responses and increased cellular exhaustion in *Hdac7^ko^* mice infected with LCMV

**Figure 5:**
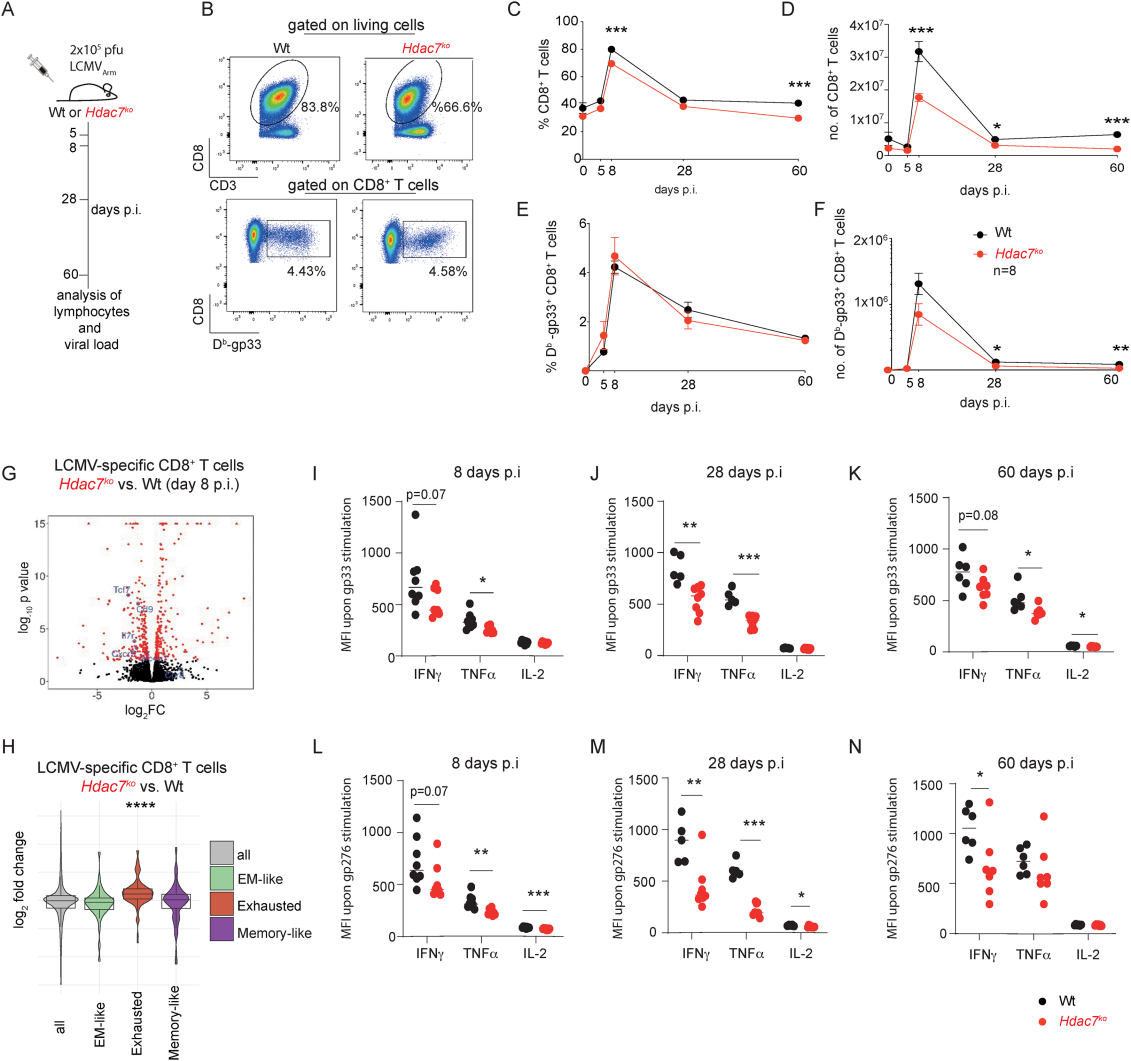
HDAC7 controls maintenance of CD8^+^ T cells and prevents cellular exhaustion during repeated LCMV infection. **(A)** Wt or *Hdac7^ko^* mice were infected with 2x10^5^ plaque-forming units (pfu) LCMV-Armstrong (LCMVArm) virus by i.p. injections. Splenocytes from infected mice were analyzed by flow cytometry 5, 8, 28, and 60 days post-infection (p.i.). **(B)** Representative plots gated on CD3^+^ T cells (upper panel) displaying the frequency of CD8^+^ T cells and D^b^-Gp33-streptamer^+^ CD3^+^CD8^+^ T cells (lower panel) of LCMV_Arm_-infected Wt and *Hdac7^ko^* mice 8 days p.i. **(C–F)** Frequencies (left) and total counts per spleen (right) of CD3^+^CD8^+^D^b^-Gp33-streptamer^+^ T cells at various time points p.i. (n=8, multiple t test). **(G)** Volcano plots showing differentially regulated genes compared to Wt from comparative RNA-sequencing analysis of LCMV-specific CD8^+^ T 8 days after LCMV infection (n=3). **(H)** *In silico* enrichment analysis of published gene sets^33^ obtained from tumor infiltrating CD8^+^ T cells in LCMV-specific CD8^+^ T cells 8 days after LCMV infection isolated from *Hdac7^ko^* mice when compared to the respective Wt. **(I–K)** Mean fluorescence intensity (MFI) of IFNy, TNFα, and IL-2 in Wt and *Hdac7^ko^* CD8^+^ T cells after stimulation with D^b^-Gp33-peptide. **(L–N)** MFI of IFNy, TNFα and IL-2 in Wt and *Hdac7^ko^* CD8^+^ T cells after stimulation with gp276-peptide on day 8, day 28 and day 60 p.i with LCMV_Arm_ (day 8 p.i.: n= 7-8; day 28 p.i.: n= 5-8; day 60 p.i.: 6–7 animals per group, multiple t test). *p < 0.05, **p < 0.01, ***p < 0.001.

In case of chronic and repetitive antigen stimulation, CD8^+^ T cells progress into an exhausted phenotype at the expense of proper memory differentiation and responses (14, 41). Following up on the phenotypic differences of CD8^+^ T cells that we observed during our *in vivo* tumor experiments, we decided to functionally compare CD8^+^ T cell responses in *Hdac7^ko^* and Wt mice over the course of acute and recurring viral infections with LCMV as a second model to understand the role of HDAC7 in CD8^+^ T cell-dependent immune responses against viruses. To this end, we first analyzed CD8^+^ T cell responses in *Hdac7^ko^* mice and Wt littermates during acute infections with LCMV Armstrong (LCMV_Arm_) (**Figure 5A**). As shown in **Figures 5B–D**, both the absolute number and the frequencies of total CD8^+^ T cells were significantly lower in *Hdac7^ko^* mice compared to Wt littermates at all timepoints of infection including steady-state conditions. The frequencies of LCMV-specific D^b^-Gp33^+^CD8^+^ T cells were not changed in *Hdac7^ko^* mice upon LCMV infection suggesting that the initial development of short lived D^b^-Gp33^+^ effector CD8^+^ T cells was unaffected by HDAC7-deficiency (**Figure 5E**). However, in line with a general decrease in total CD8^+^ T cell numbers in *Hdac7^ko^* mice, the absolute numbers of LCMV-specific D^b^-Gp33^+^CD8^+^ T cells were significantly reduced in *Hdac7^ko^* mice compared to Wt littermates 28 and 60 days post LCMV infection (**Figure 5F**). Despite a lower absolute number of virus-specific CD8^+^ T cells, comparable copy numbers of LCMV_Arm_ could be detected in the serum of virus-infected *Hdac7^k^*^o^ mice and Wt littermates by quantitative (q)PCR (**Supplementary Figure 6A**), highlighting that the initial effector functions of CTL are preserved in *Hdac7^ko^* mice, despite an increased Eomes/Tbet ratio in *Hdac7^ko^* mice (**Supplementary Figures 6B–E**). These observations suggest a deterred transcriptional programming of *Hdac7*-deficient CD8^+^ T cells due to increased Eomes expression which has been described as a transcriptional marker of CD8^+^ T cell exhaustion (19, 50, 51). Accordingly, comparative RNA-sequencing of LCMV-specific gp33^+^ CD8^+^ T cells isolated from LCMV-infected *Hdac7^ko^* mice revealed an altered transcriptome compared to Wt littermates (**Figure 5G**). When assessing the enrichment of published gene sets implicated in the development and function of LCMV-specific CD8^+^ memory T cells (41), we found that *Hdac7*-deficient, virus-specific CD8^+^ T cells displayed a significant enrichment of differentially expressed genes related to apoptosis, metabolism, transcription, signaling, effector functions, glycosylation, cell homing and the expression of cell surface receptors (**Supplementary Figures 2F, G**). Similar to *in vitro* activated *Hdac7^ko^* CD8^+^ T cells and our observation in tumors, we furthermore noted a significant enrichment of published gene-signatures associated with the exhaustion of tumor-infiltrating CD8^+^ T cells in the transcriptome of LCMV-specific *Hdac7*-deficient CD8^+^ T cells (44) (**Figure 5H**), further supporting that HDAC7 regulates cellular exhaustion of CD8^+^ T cells in anti-viral and anti-tumor immunity.

To analyze if the changes in cell differentiation observed in *Hdac7*-deficient CD8^+^ T cells during viral infections would also be reflected in altered cytokine patterns, we subsequently compared the production of IFNγ, TNFα and IL-2 in splenocytes of LCMV_Arm_-infected *Hdac7^ko^* and Wt mice upon *ex vivo* stimulation with the immunodominant LCMV peptide Gp33 and the immune-subdominant Gp276 peptide (52, 53). In line with the pre-activated state of CD8^+^ T cells in *Hdac7^ko^* mice under steady-state conditions, stimulation with Gp33 peptide induced a significantly higher frequency of IFNγ and TNFα producing cells in *Hdac7*-deficient CD8^+^ T cells 5 days p.i. but an equivalent fraction of IFNγ, TNFα and IL-2 producing cells 8 days p.i. (**Supplementary Figures 7A–C**). Upon Gp276 stimulation, *Hdac7^ko^* mice displayed decreased frequencies of all three cytokines for all timepoints we analyzed (**Supplementary Figures 7D–F**). Moreover, in line with an exhaustion-like phenotype, the capacity to produce cytokines were severely impaired in *Hdac7^ko^* CD8^+^ T cells upon *ex vivo* re-stimulation with both the Gp33 and the Gp276 peptides as HDAC7-deficient T cells displayed significantly reduced mean fluorescence intensities (MFI) of IFNy, TNFα and IL-2 (**Figures 5I–N**), which is considered as a key feature of T cell exhaustion (54). Additionally, a significant reduction in the total number of cytokine-producing cells was detected at almost all indicated time points, which can be explained by an additive effect of a lower frequency and absolute numbers of CD8^+^ T cells (**Supplementary Figures 7G–L** & **10**).

**Figure 6:**
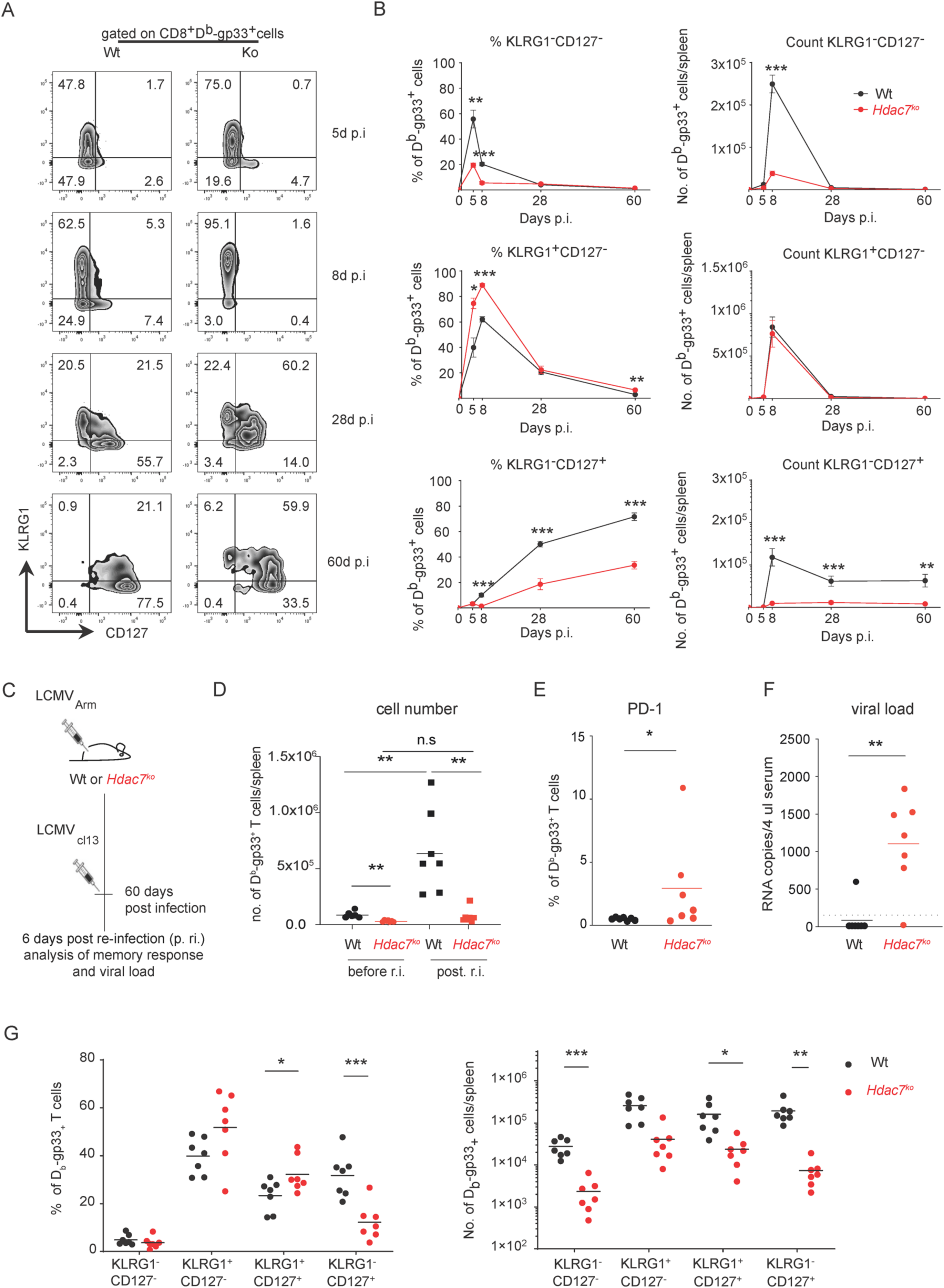
*Hdac7^ko^* CD8^+^ T cells show impaired memory formation during LCMV_Arm_ infection and failed memory recall after LCMV_cl13_ re-infection. **(A)** Representative density blots of the cytometric analysis of antigen specific CD3^+^CD8^+^ splenocytes regarding KLRG1 and CD127 expression at the indicated timepoints during LCMV_ARM_ infection. **(B)** Frequencies and absolute numbers of double negative cells, SLEC (short lived effector cells KLRG1^+^CD127^-^) and MPEC (memory precursor effector cells, KLRG1^–^CD127^+^) at the indicated time points during LCMV_ARM_ infection. **(C)** 60 days after primary infection with LCMV_Arm_, Wt or *Hdac7^ko^* mice were re-challenged with LCMV clone 13 (LCMV_cl13_). On day 6 post re-infection splenocytes were analyzed by flow cytometry. **(D)** Total counts per spleen of CD3^+^CD8^+^D^b^-Gp33-streptamer^+^ T cells in Wt and *Hdac7^ko^* mice before and after LCMV_cl13_ re-infection (n=6-7, multiple t test). **(E)** Dot plots showing the frequency of PD-1^+^CD3^+^CD8^+^D^b^-Gp33-streptamer^+^ T cells in Wt and *Hdac7^ko^* mice 6 days p.r.i. assessed by flow cytometry (n=7 per group, multiple t test). **(F)** Viral load in serum of LCMV_Arm_-infected Wt and *Hdac7^ko^* mice after re-infection with LCMV_cl13_ (n=7, multiple *t*-test). **(G)** KLRG1 and CD127 analysis of CD3+CD8+Db-Gp33-streptamer+ splenocytes of LCMVARM infected Wt or *Hdac7^ko^* mice, reinfected with LCMV^Cl13^ at day 60 and assessed by flow cytometry at day 6 post re-infection. (n=8-9, multiple t test); *p<0.05, **p<0.01, ***p<0.001.

Until now, we had observed an increased expression of exhaustion markers in *Hdac7^ko^* CD8^+^ T cells during repetitive stimulation *in vitro* as well as during chronic antigen exposure in tumors. Another important factor in CD8+ T cell function and fitness is the formation of an effective memory compartment. Therefore, we longitudinally characterized the expression of memory markers in mice infected with LCMV_Arm_ virus reaching day 60. As shown in **Figures 6A, B** we observed that antigen-specific CD8^+^ T cells from *Hdac7^ko^* mice did not transition from the KLRG1^+^ state to KLRG1^-^CD127^+^ memory precursor effector cells (MPEC), resulting in significantly lower absolute numbers of virus-specific memory cells. To better understand how *Hdac7^ko^* CD8^+^ T cells respond to repeated antigen exposure *in vivo*, we next analyzed the efficacy of *Hdac7^ko^* mice to handle secondary infections after prior immunization. Thus, Wt or *Hdac7^ko^* mice were infected with LCMV_Arm_ and subsequently challenged with LCMV clone 13 (LCMV_cl13_) 60 days after initial infection. Spleens of infected mice were then harvested 6 days post re-infection (p.ri.) with LCMV_cl13_ (**Figure 6C**) While D^b^-Gp33^+^ Wt CD8^+^ T cells expanded 9 fold upon re-exposure to virus, *Hdac7^ko^* CD8^+^ T cells expanded only 3-fold upon antigen re-encounter (**Figure 6D**). Moreover, D^b^-Gp33^+^ *Hdac7^ko^* cells displayed a significantly higher expression of the exhaustion marker PD-1 (**Figure 6E**), which was paralleled by a significantly higher viral load 6 days post re-infection (**Figure 6F**), which we attribute to the diminished MPEC fraction (**Figure 6G**). These results further underscore that HDAC7 is required to suppress cellular exhaustion in CD8^+^ T cells and to maintain CD8^+^ T cell dependent immune responses upon repetitive antigen-exposure during both anti-tumor and anti-viral immunity.

## Discussion

Taken together, we here identified HDAC7 as a crucial regulator of apoptosis, Ca^2+^ homeostasis, immune metabolism and cellular exhaustion, that is required for CD8^+^ T cell-dependent anti-viral and anti-tumor immunity. Thus, we not only observed that HDAC7 is critical for anti-tumor immunity against lymphoma cells but that it is furthermore pivotal for the proper development and maintenance of virus-specific memory CD8^+^ T cells and subsequently for effective memory recall responses.

Unexpectedly, we observed a pre-activated phenotype under steady state conditions in CD8^+^ T cells and a transcriptional upregulation of several exhaustion markers including Tim-3, PD-1, Tigit, Ctla-4, and Lag-3 upon stimulation of *Hdac7^ko^* CD8^+^ T cells. Furthermore, *Hdac7^ko^* mice featured a significantly reduced frequency of CD8^+^ T cells, which was paralleled by an increased amount of apoptosis in *Hdac7*-deficient CD8^+^ T cells upon activation *in vitro*. Accordingly, we observed a transcriptional deregulation of several pro-apoptotic genes in CD8^+^ T cells of *Hdac7^ko^* mice, which was in line with previous findings in T cells expressing an inactive mutant of *Hdac7*. Here, CD8^+^ T cells displayed a deregulation of apoptosis in the thymus and subsequently an impaired thymic selection of T cells in a Fas/FasL- independent manner (29). Our findings indicate that HDAC7 promotes FasL-dependent apoptosis in peripheral CD8^+^ T cells by regulating MEF2D binding to the *Fasl* promoter, as shown by MEF2D-ChIP assays, consistent with the established interaction between HDAC7 and MEF2 transcription factors (32, 34, 55). While loss of HDAC7 is associated with differential histone acetylation at multiple genomic sites, no such change occurs at the *Fasl* locus, pointing to the transcription factor–binding component of HDAC7’s dual role, rather than its chromatin-modifying activity, as the mechanism underlying the pro-apoptotic phenotype. We also observed increased apoptosis of *Hdac7^ko^* CD8^+^ T cells upon repetitive stimulation *in vitro*, paralleled by an increased expression of key exhaustion markers including PD-1, Tim-3 and Tigit. Adoptive transfer experiments in tumor bearing mice also revealed that lack of HDAC7 both impairs the persistence of CTLs *in vivo* as well as the suppression of PD-1 and Tim-3 expression by CTLs in the tumor microenvironment, which are the hallmarks of CD8^+^ T cell exhaustion, further implying that HDAC7 is a crucial factor to control the exhaustion and survival of CD8^+^ T cells. Sukumar et al. (56) and Perl et al. (57) previously showed that CD8^+^ T cells with high mitochondrial membrane potential (ΔΨm) often associated with a KLRG1^high^ phenotype, exhibit features of terminal effector differentiation, including poor expansion and increased susceptibility to Fas-mediated apoptosis, although recent studies indicate that KLRG1^high^ populations can retain functional plasticity (4). Our findings match these observations, as we also detect enhanced mitochondrial membrane potential and compromised anti-tumor function of *Hdac7*^ko^ CD8^+^ T cells *in vivo*, along with increased Fas-mediated apoptosis. These results suggest that elevated ΔΨm contributes to the decreased survival and impaired function of *Hdac7^ko^* CD8^+^ T cells in our tumor and viral-infection model.

While *Hdac7* deficiency impairs SOCE-mediated calcium signaling, the increased mitochondrial membrane potential and glutamine uptake do not indicate improved metabolic fitness but rather an altered metabolic state associated with activation and differentiation. In this context, these changes are consistent with a shift toward a more differentiated, short-lived phenotype with reduced survival and function.

In addition, we observed increased expression of several glutamine receptors and enhanced absolute glutamine uptake in *Hdac7^ko^* CD8^+^ T cells. Notably, pre-incubation of tumor-specific CD8^+^ T cells in glutamine-free media or in presence of glutamine metabolism inhibitors has been shown to enhance memory T cell markers, leading to improved survival, reduced exhaustion and increased T cell infiltration after adoptive transfer into tumor-bearing mice (58, 59). Moreover, *in vivo* application of glutamine antagonist skews CD8^+^ T cells and TILs toward long-lived memory-like subsets with less exhausted, anergic phenotype and enhanced anti-tumor activity (60). In contrast, *Hdac7^ko^* CD8^+^ T cells, which exhibit elevated expression of glutamine transporters, display increased cell death, higher exhaustion markers expression, and impaired anti-tumor functionality, suggesting that HDAC7 deficiency contributes to this phenotype by modulating glutamine metabolism upon activation.

Astonishingly, the CD4^+^ T cell compartment of LCMV-infected *Hdac7^ko^* mice was largely unaffected and no significant differences in the colitogenic potential of HDAC7-deficient naive CD4^+^ T cells could be detected as assessed in a T-cell transfer model of colitis. Therefore, CD4^+^ T cells can sufficiently provide help to CD8^+^ T cells, suggesting that the observed defects in anti-viral immunity and anti-tumor immunity in *Hdac7^ko^* mice are due to CD8^+^ T cell intrinsic functions of HDAC7. We also found that HDAC7 is also required for the function of memory CD8^+^ T cells. Thus, *Hdac7*-deficient CD8^+^ T cells revealed a defective cytokine production of TNFα, IFNγ and IL-2. Importantly, *Hdac7*-deficient memory T cells failed to give rise to effector CD8^+^ T cells upon re-infection with LCMV_cl13_, which resulted in a defective viral clearance and an induction of the cellular exhaustion marker PD-1 in virus-specific CD8^+^ T cells of *Hdac7^ko^* mice. Furthermore, virus-specific *Hdac7^ko^* CD8^+^ T cells displayed an impaired cellular expansion as well as a significantly decreased total number in *Hdac7^ko^* mice upon re-infection with LCMV_cl13_. These observations are also in line with the exhaustion-like phenotype of *Hdac7^ko^* CD8^+^ T cells during both *in vitro* stimulations and chronic antigen exposure within tumors in which *Hdac7^ko^* CD8^+^ T cells cannot handle repetitive stimulations properly due to an increased expression of exhaustion markers and impaired *in vivo* persistence.

In line with these functional impairments, *Hdac7*-deficient CD8^+^ T cells were characterized by a reduced Ca^2+^ influx and a transcriptional deregulation of several SOCE-signaling components. The latter has been described to play a pivotal role not only in the regulation of T cell metabolism (13) but also in the function and maintenance of CD8^+^ T cell-dependent memory responses (12). Thus, *Stim1^fl/fl^Stim2^fl/f^CD4-Cre* mice display a similar phenotype as *Hdac7^ko^* mice upon viral infection with LCMV as they feature exhausted CD8^+^ T cells with high PD-1 and Tim-3 expression as well as impaired production of IL-2, TNFα and IFNγ, despite an elevated Eomes expression (12). Additionally, they are also incapable of mounting functional recall responses against reinfection with LCMV_cl13_ and show severe defects in anti-tumor immune responses in mouse models of melanoma and lymphoma (10).

While these findings suggest potential implications for human disease and therapeutic interventions, they are derived from preclinical models and therefore require validation in human systems, which represents an important direction for future studies. Previous studies have demonstrated that altered HDAC7 function can affect thymic development and T cell selection, which may contribute to the reduced naïve CD8^+^ T cell compartment observed at steady state. This raises the possibility that some of the effects observed in *in vivo* models, where Hdac7-deficient mice are compared to WT mice, may in part reflect differences in the CD8^+^ T cell repertoire or subset composition at baseline. However, for the *in vitro* activation followed by RNA-sequencing analyses, naïve CD8^+^ T cells were flow-sorted prior to stimulation, allowing comparison from a matched starting population. In addition, early effector and antigen-specific CD8^+^ T cell responses during acute infection were comparable, suggesting that the steady-state imbalance does not fully account for the functional phenotypes observed in memory and exhaustion-related settings.

In regard to the critical function of HDAC7 for the survival of functional CD8^+^ T cells in mice and the observation that HDAC7 controls the expression of exhaustion markers including *Pdcd1, Havcr2*, *Tigit*, *Ctla4* and *Lag3* in murine CD8^+^ T cells, downregulation or functional inhibition of HDAC7 might represent an escape mechanism for human tumors and pathogens resulting in impaired anti-tumor and anti-viral immunity. The dependence of anti-viral memory responses and anti-tumor immune functions on HDAC7 has critical implications for the clinical evaluation of pharmacologic HDAC inhibitors that are currently being tested for the treatment of autoimmune diseases and various types of cancer (61). Thus, the pan-HDAC inhibitors vorinostat and panobinostat were shown to decrease IFNγ production, the survival and the cytotoxic capacity of human HIV-specific CD8^+^ T cells (62). Thereby, HDAC inhibitors do not only block the enzymatic activity of HDAC7 but suppress the expression of HDAC7 as shown for various cell types including fibroblasts, epithelial cells, bladder and prostate cancer cells as well as myeloma cells (63). Likewise, we observed a downregulation of HDAC7 after treatment of CD8^+^ T cells with vorinostat (**Supplementary Figures 9A, C**), significantly reducing the protein expression of HDAC7 and triggering the induction of apoptosis (64). Moreover, we observed an increase in the acetylation of H3 at lysine 9 (K9) and K14 residues at the global level as well as locus-specific changes, including at several metabolism/apoptosis-related genes in *Hdac7^ko^* CD8^+^ T cells suggesting that HDAC7 might regulate some processes through histone deacetylation. In light of the growing number of HDAC inhibitors that are currently tested for the treatment of autoimmune and neoplastic diseases, our findings caution that inhibition of HDAC7 interferes with the development and maintenance of appropriate memory responses e. g., upon vaccination and might thus alter the function, the metabolic fitness and the survival of CD8^+^ T cells during immune responses against viral infections and tumors.

## Data Availability

Sequencing data have been uploaded to the European Nucleotide Archive (https://www.ebi.ac.uk/ena/browser/home) and can be accessed as PRJEB111931 (mRNA sequencing) and PRJEB111947 (ChIP sequencing).
