# Supplementary material for: HDAC7 controls anti-viral and anti-tumor immunity by CD8+ T cells
Source: Front Immunol. 2026 May 12;17:1816695. doi: 10.3389/fimmu.2026.1816695 (PMC13201152; doi:10.3389/fimmu.2026.1816695)
Supplement: Supplementary file 1 [file Image1.pdf]

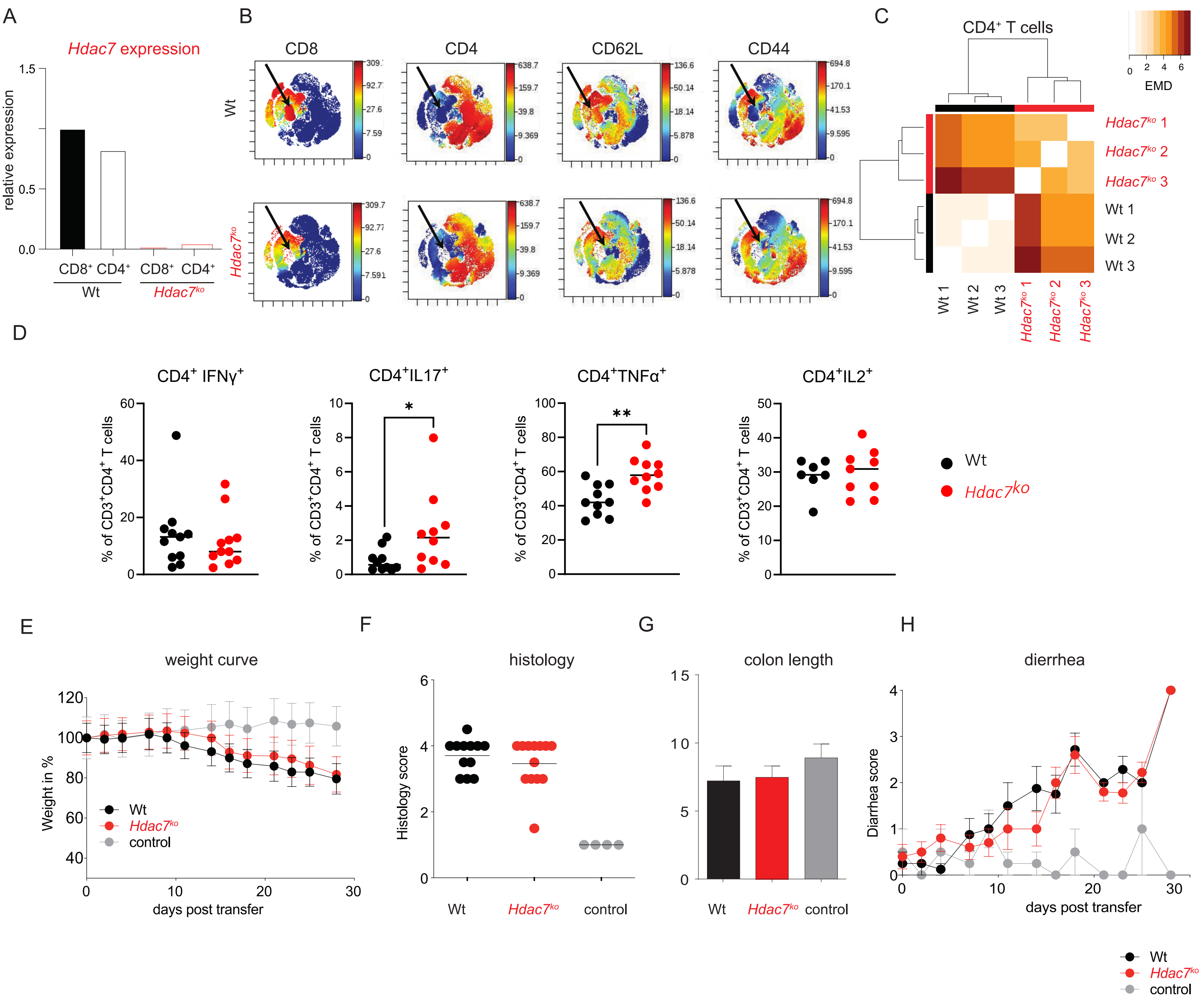

Supplementary Figure 1: *Hdac7$^{fl/fl}$CD4-Cre* mice have a preactivated phenotype of $CD8^+$ T cells and a largely unaffected $CD4^+$ T cell compartment. (A) Deletion efficiency of *Hdac7* in *Hdac7$^{fl/fl}$CD4-Cre* (*Hdac7^{ko}*) mice. $CD4^+$ and $CD8^+$ T cells were isolated from spleens of *Hdac7^{ko}* mice and Wt littermates. qPCR was performed to check the deletion efficiency of *Hdac7*. *Hdac7* expression was normalized to β-actin expression. Representative column plots of two independent experiments performed with two biologically independent samples per group. **(B)** Representative 2D-tSNE analyses of mass cytometry data comparing the distribution of CD8, CD4, CD62L and CD44 expressing cells in pre-gated $CD45^+CD3^+$ T cells isolated from the spleens of Wt littermates or *Hdac7$^{fl/fl}$-Cd4-Cre* (*Hdac7^{ko}*) mice (n=3 for both groups). **(C)** Heatmap displaying the pairwise earth-mover's distance (EMD) values of the cellular density distribution within $CD4^+$ T cell population after PMA/ionomycin stimulation over a 2D t-SNE space. **(D)** Isolated splenocytes of healthy *Hdac7^{ko}* mice and WT mice, stimulated with PMA/Ionomycin were stained intracellularly for the indicated cytokines and cells were analyzed by flow cytometry. (n=7 – 11, multiple t-test, $*p < 0.05$, $**p < 0.01$) **(E-G)** Transfer colitis experiments. $4x10^5$ naïve $CD4^+$ T cells from Wt or *Hdac7^{ko}* mice were i.p. injected into *Rag2$^{-/-}$* mice to induce transfer colitis. Control *Rag2$^{-/-}$* mice were injected with PBS only. Mice were weighed and scored every second day and sacrificed on day 28 post transfer. **(E)** Line graphs showing the percentage of weight changes over the time of the disease. **(F)** Dot plots displaying the histology score composed of cell infiltration and tissue damage. **(G)** Bar graphs summarizing the colon length in cm. **(H)** Line graphs depicting the diarrhea score assessed by the consistency of feces.

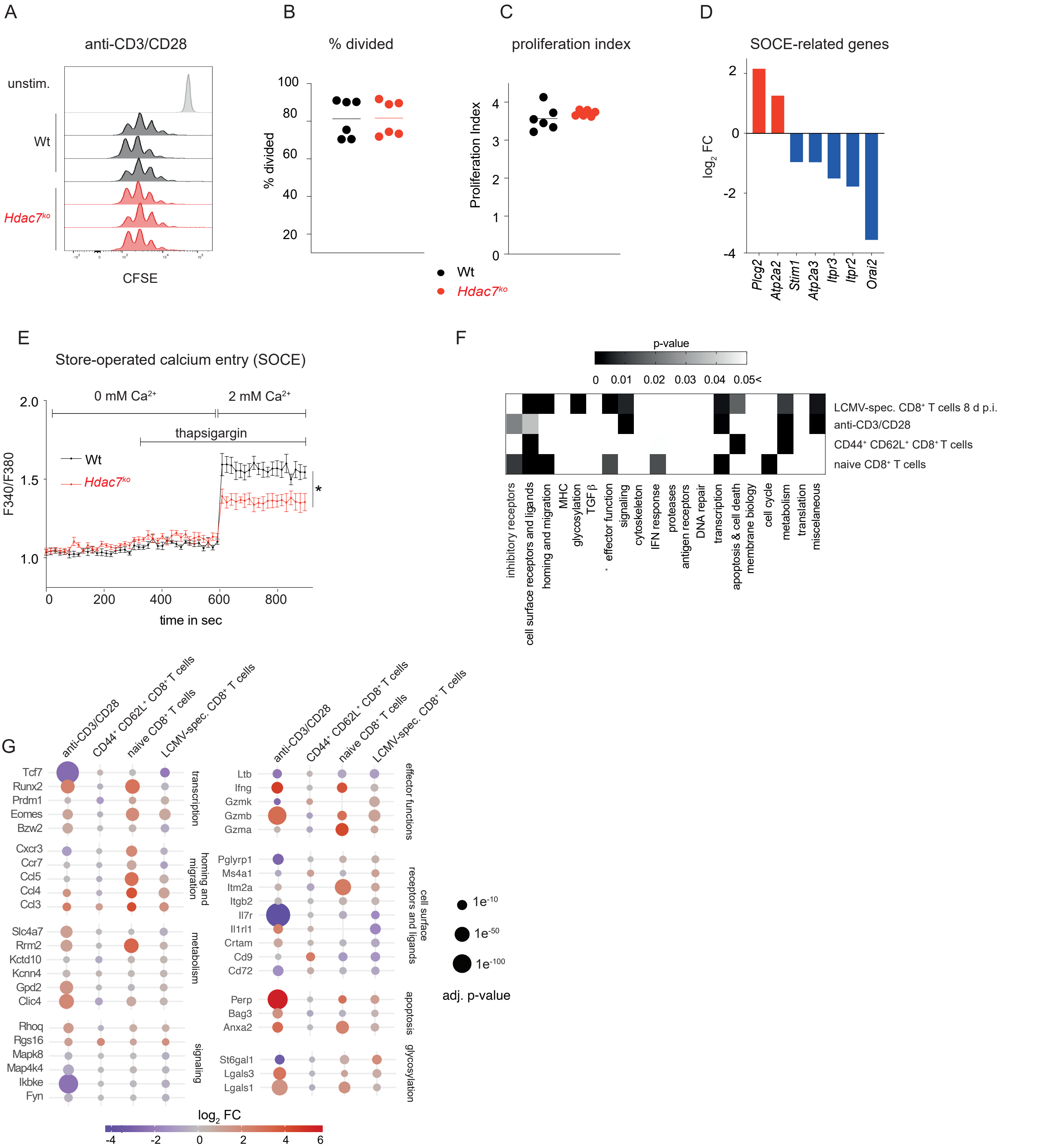

Supplementary Figure 2: HDAC7 deletion in CD8+ T cells results in disturbed Store-Operated Calcium Entry (SOCE) and amino acid metabolism. (A) Representative histograms of CFSE staining measured by flow cytometry 3 days post *in vitro* anti-CD3/CD28 activation of Wt and *Hdac7ko* CD8+ T cells (two independent experiments, n=6). **(B)** Dot plots showing the percentage of divided cells, and **(C)** the proliferation index during CFSE dilution assay (n=6). Percentage of divided cells and the proliferation index were calculated by FlowJo software. **(D)** Fold-change expression of SOCE-component genes from comparative RNA-sequencing of *Hdac7ko* CD8+ T cells activated with anti-CD3/CD28 antibodies for 48 h compared to the respective Wt (for all $p<0.05$, n=3 per group). **(E)** Calcium influx in Wt and *Hdac7ko* CTLs, mean ± SEM of Ca^{2+} influx rates (n=3 independent experiments, performed in duplicates, multiple t test). (F) Heatmaps showing the statistically significant enrichment of differentially regulated biological pathways in the indicated subsets of *Hdac7ko* CD8+ T cells compared to the respective Wt, that have been previously implicated in the development and function of LCMV-specific CD8+ memory T cells[7], and (G) top ten genes deregulated in these biological pathways in anti-CD3/CD28 activated, CD44+CD62L+, naïve and LCMV-specific CD8+ *Hdac7ko* CD8+ T cells compared to the respective Wt.

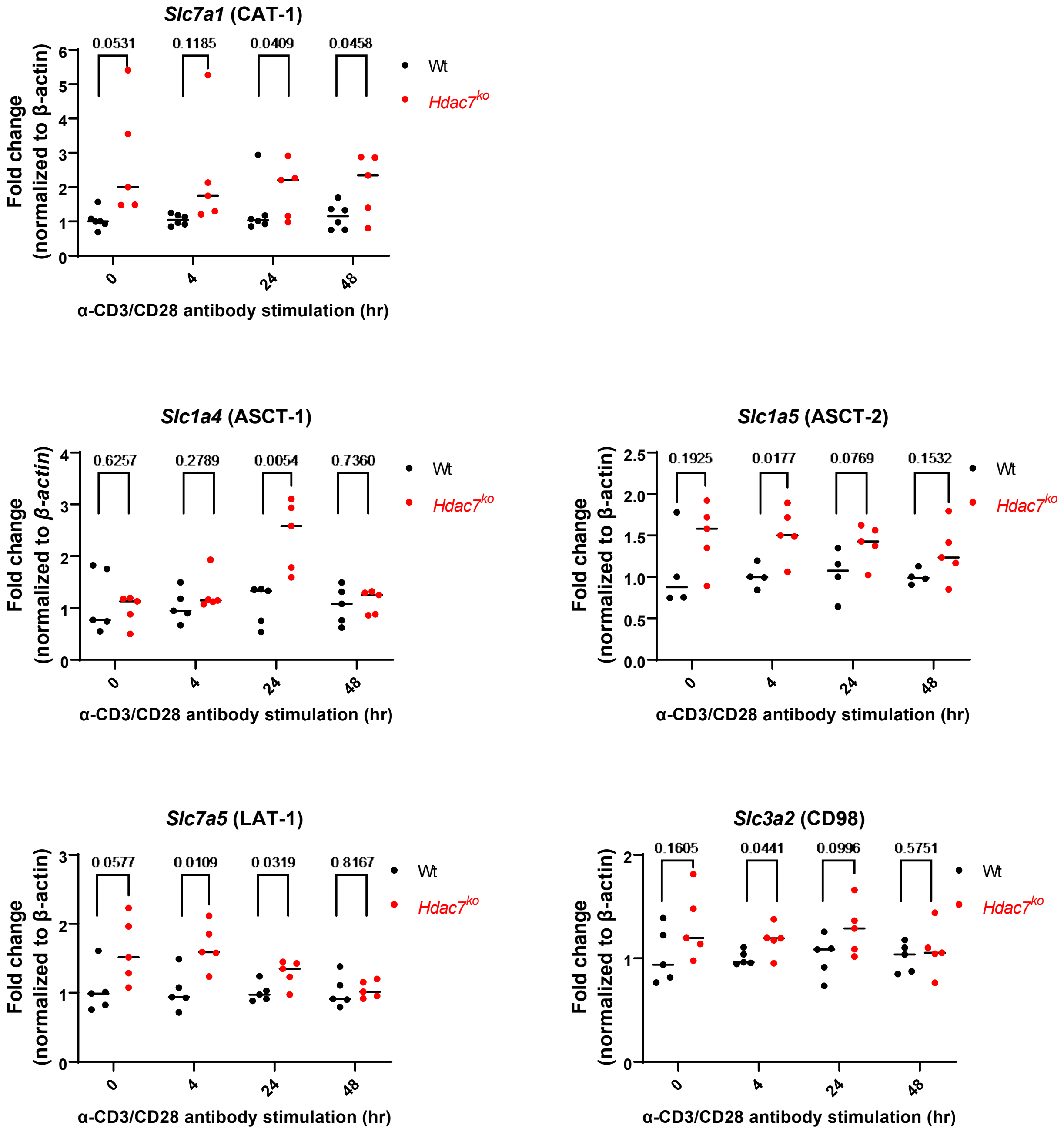

Supplementary Figure 3: HDAC7 regulates expression levels of several amino acid transporters at RNA level in CD8⁺ T cells. Differential expression derived from RT-qPCR results of selected amino acid transporters in Wt and *Hdac7*^{ko} CD8$^+$ T cells stimulated for indicated time. ΔCT values of target genes were normalized to β-actin mRNA, whereas ΔΔCT values were generated by subtracting the average ΔCT values of Wt group from the individual ΔCT values of each sample. Fold change was generated by normalizing ΔΔCT values of all data points to the average ΔΔCT value of Wt group of each experimental batch. Two to three independent experiments, n=4-6, technical duplicate, unpaired t test.

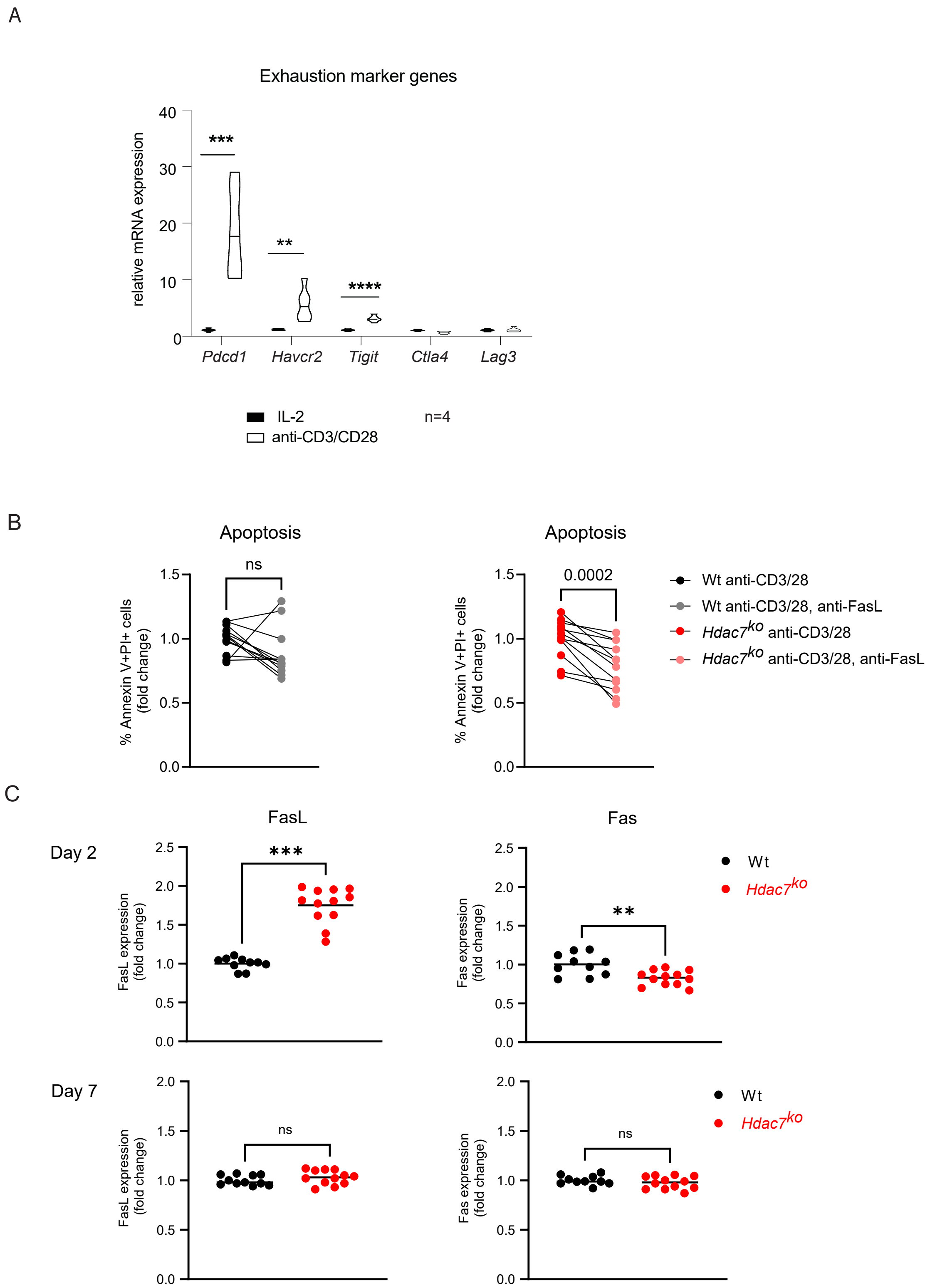

Supplementary Figure 4: *Hdac7^{ko}* CD8$^+$ T cells have increased apoptosis during chronic *in vitro* stimulation. (A) Violin plots showing the expression of exhaustion marker genes *Pdcd1*, *Havcr2*, *Tigit*, *Ctla4* and *Lag3* in Wt CD8$^+$ T cells exposed to anti-CD3/CD28 stimulation or grown in the presence of IL-2 without repeated stimulation. Ct values were normalized to the Ct values of 36b4 and $2^{-\Delta Ct}$ was calculated. The expressions are shown relative to IL-2 controls. (n=4, multiple t test). **(B)** Apoptosis measured by Annexin V, propidium iodide (PI) staining followed by flow cytometry analysis were performed in Wt and *Hdac7^{ko}* CD8$^+$ T cells with or without anti-FasL antibody supplement. Fold change was generated by normalizing the frequency of AnnexinV$^+$PI$^+$ of all data points to the average value of Wt group of each experimental batch. (four independent experiments, n=10-12, technical duplicates, unpaired t test) **(C)** Expression level of FasL and Fas in Wt and *Hdac7^{ko}* CD8$^+$ T cells activated for 2 or 7 days was measured via flow cytometry. Fold change was generated by normalizing gMFI values of all data points to the average value of Wt group of each experimental batch. Four independent experiments. (n=10-12, technical duplicates, unpaired t test, **p<0.01, ***p<0.005)

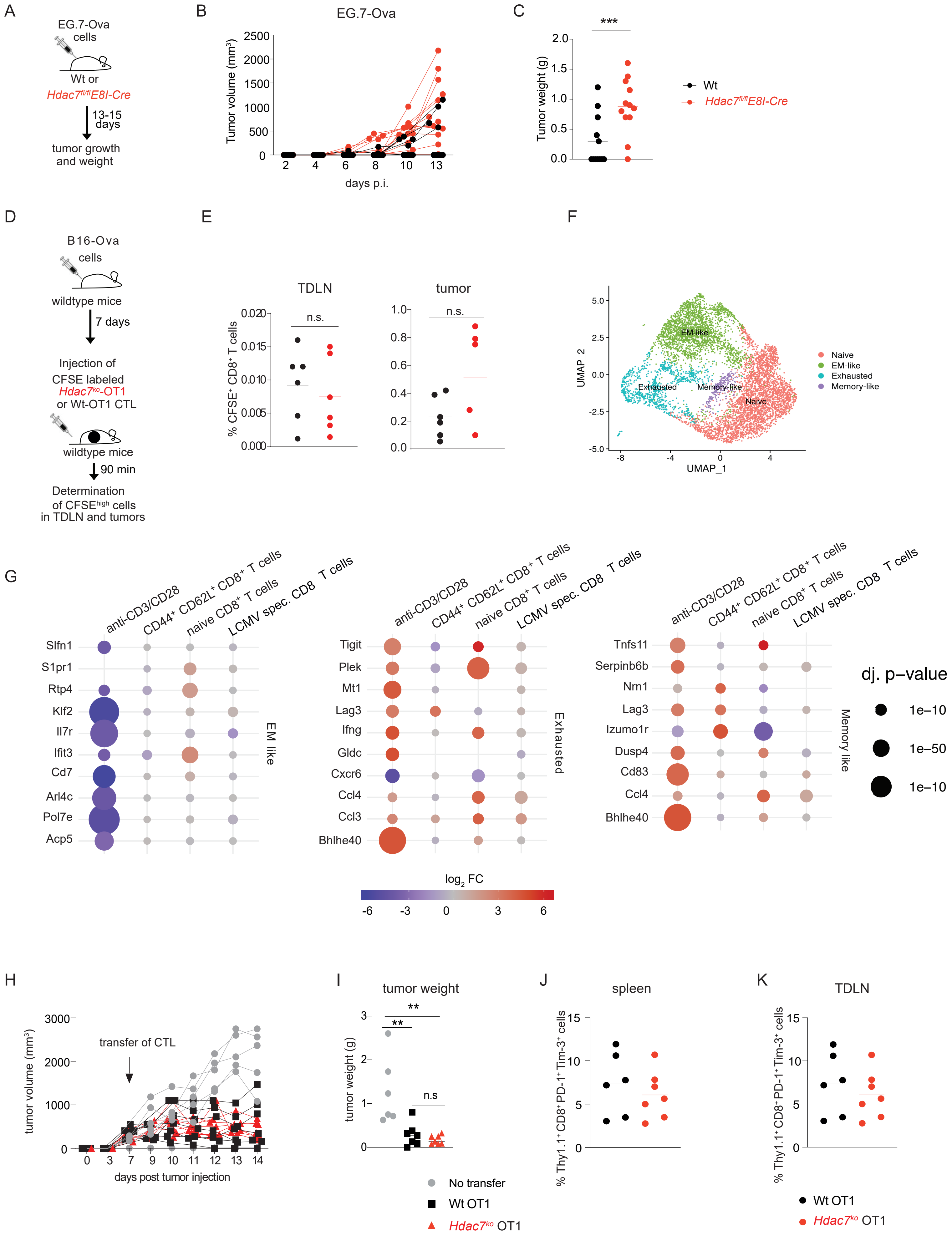

A
EG.7-Ova cells
Wt or
Hdac7fl/flE8I-Cre
13-15 days
tumor growth and weight
B
EG.7-Ova
Tumor volume (mm3)
days p.i.
C
***
Tumor weight (g)
Wt
Hdac7fl/flE8I-Cre
D
B16-Ova cells
wildtype mice
7 days
Injection of CFSE labeled Hdac7ko-OT1 or Wt-OT1 CTL
wildtype mice
90 min
Determination of CFSEhigh cells in TDLN and tumors
E
TDLN
tumor
n.s.
n.s.
% CFSE+ CD8+ T cells
F
UMAP_2
UMAP_1
EM-like
Exhausted
Memory-like
Naive
Naive
EM-like
Exhausted
Memory-like
G
anti-CD3/CD28
CD44+ CD62L+ CD8+ T cells
naive CD8+ T cells
LCMV spec. CD8 T cells
Slfn1
S1pr1
Rtp4
Klf2
Il7r
Ifit3
Cd7
Arl4c
Pol7e
Acp5
EM like
Tigit
Plek
Mt1
Lag3
Ifng
Gldc
Cxcr6
Ccl4
Ccl3
Bhlhe40
Exhausted
Tnfs11
Serpinb6b
Nrn1
Lag3
Izumo1r
Dusp4
Cd83
Ccl4
Bhlhe40
Memory like
dj. p-value
1e−10
1e−50
1e−10
log2 FC
H
tumor volume (mm3)
transfer of CTL
days post tumor injection
I
tumor weight
**
**
n.s
tumor weight (g)
No transfer
Wt OT1
Hdac7ko OT1
J
spleen
% Thy1.1+ CD8+ PD-1+ Tim-3+ cells
K
TDLN
% Thy1.1+ CD8+ PD-1+ Tim-3+ cells
Wt OT1
Hdac7ko OT1

Supplementary Figure 5: The anti-tumor immune responses of HDAC7 is $CD8^+$ T cell intrinsic. (A) Wt and *Hdac7$^{fl/fl}$E8I-Cre* mice were intradermally (i.d.) injected with $1x10^6$ EG.7-Ova cells. Tumor growth was followed for 14 days. **(B)** Tumor growth in Wt and *Hdac7$^{fl/fl}$E8ICre* mice (n=11-12). **(C)** Tumor weight in Wt and *Hdac7$^{fl/fl}$E8ICre* mice on day 14 after tumor inoculation (n=11-12 mice per group). **(D)** B16-Ova bearing Wt mice were i.v. injected with CFSE labeled Wt OT1 of *Hdac7^{ko}* OT1 CTLs and sacrificed after 90 min. The frequency of CFSE-high cells was analyzed by flow cytometry. **(E)** The frequencies of CFSE-high $CD8^+$ T cells in tumor draining lymph nodes (TDLN) and B16-Ova tumors (n=5-6, multiple t test). **(F)** Unifold manifold approximation and projection (UMAP) plots displaying different subsets of B16-Ova infiltrating $CD8^+$ T cells[3]. **(G)** Top 10 regulated genes in anti-CD3/CD28 activated, $CD44^+CD62L^+$, naïve and LCMV-specific $CD8^+$ *Hdac7^{ko}* $CD8^+$ T cells compared to the respective Wt, according to the gene sets obtained from B16-Ova infiltrating $CD8^+$ T cell subsets[3] (Wilcoxon test). **(H-K)** *Hdac7^{ko}* mice were injected (i.d.) with $1x10^6$ EG.7-Ova cells. 7 days after tumor inoculation, recipient mice were injected with $7x10^6$ *in vitro* differentiated Wt OT1 or *Hdac7^{ko}* OT1 CTLs. Tumor growth was followed for 7 days post CTL transfer. **(H)** Tumor growth and **(I)** Tumor weights in mice without transfer or transferred with Wt OT1 and *Hdac7^{ko}* OT1 CTLs (n=6-7, multiple t test). **(J)** The frequency of transferred $Thy1.1^+CD8^+PD\text{-}1^+Tim\text{-}3^+$ Wt OT1 or *Hdac7^{ko}* OT1 CTLs in spleen and **(K)** TDLN. (multiple t test **p<0.01, ***p<0.001).

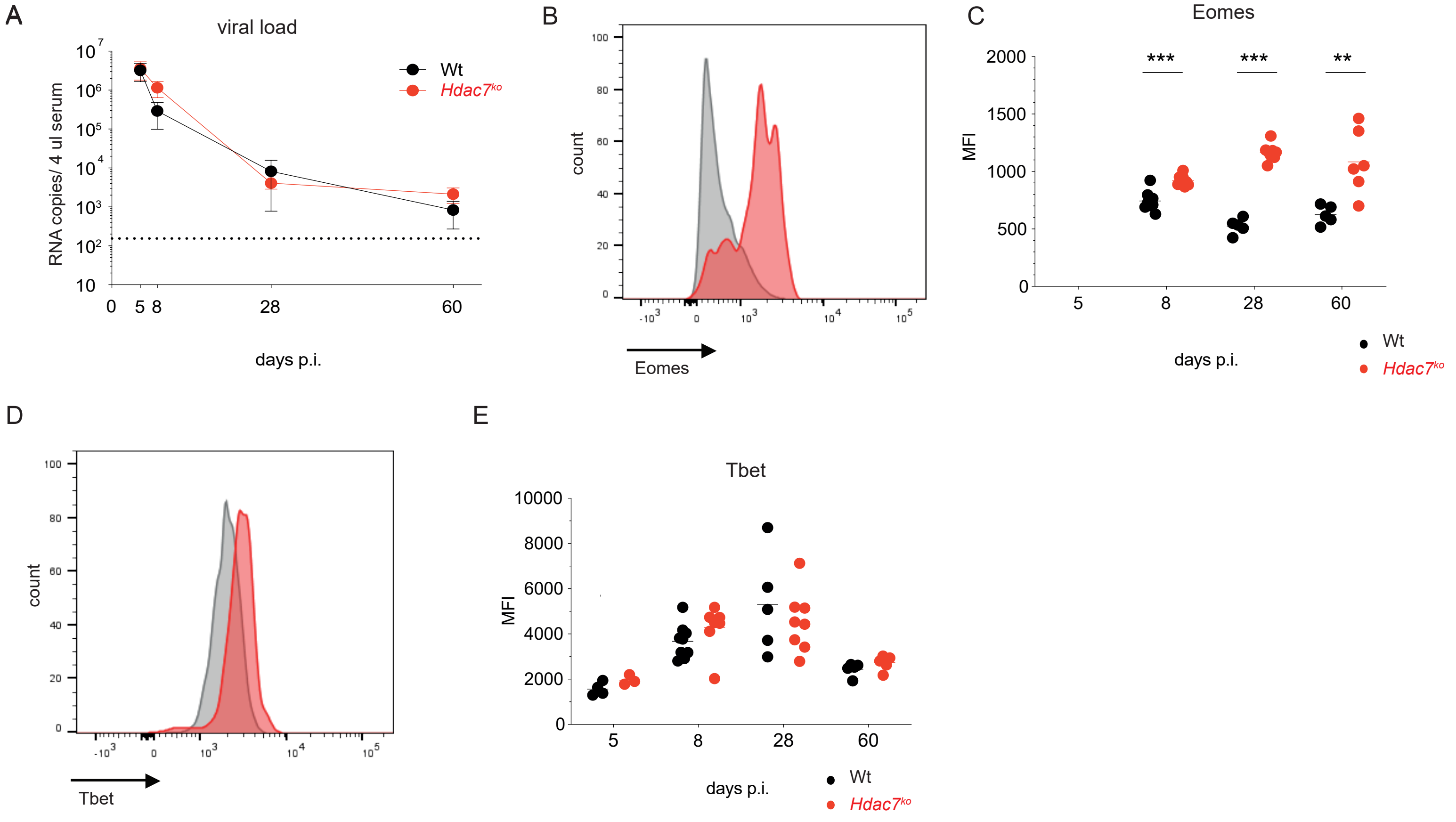

Supplementary Figure 6: *Hdac7*^{ko} $CD8^+$ T cells display increased Eomes expression during $LCMV_{Arm}$ infection. (**A**) Viral load in the serum of $LCMV_{Arm}$ infected Wt and *Hdac7*^{ko} mice as determined by RT PCR calculating $LCMV_{Arm}$ RNA copy numbers per 4 µl serum. (**B-E**) Representative histograms and dot plots displaying the mean fluorescence intensity (MFI) of (B-C) Eomes and (**D-E**) T-bet expression in $CD3^+CD8^+$Db-Gp33-streptamer$^+$ T cells isolated from the spleens of $LCMV_{Arm}$ infected Wt or *Hdac7*^{ko} mice as assessed by flow cytometry (n=8-9, multiple t test); *p<0.05, **p<0.01, ***p<0.001.

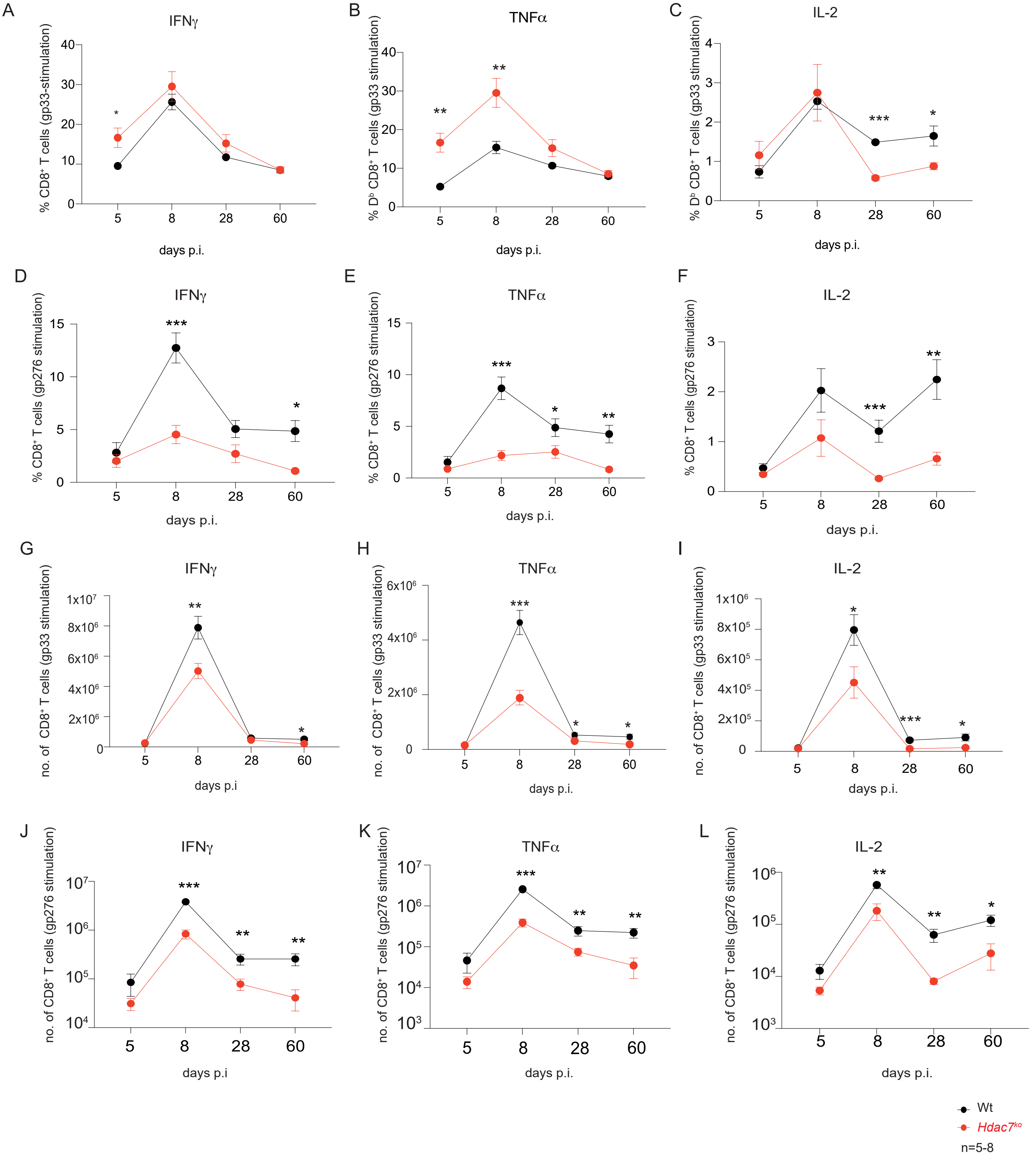

Supplementary Figure 7: *Hdac7^ko* mice have altered frequencies of cytokine producing CD8⁺ T cells during LCMV infection. (A-C) The frequencies of IFNγ, TNFα and IL-2 producing Db-gp33 peptide stimulated Wt and *Hdac7^ko* CD8⁺ T cells over the course of $LCMV_{Arm}$ infection. **(D-F)** The frequencies of IFNγ, TNFα and IL-2 producing gp276 peptide stimulated Wt and *Hdac7^ko* CD8⁺ T cells over the course of $LCMV_{Arm}$ infection. **(G-I)** Cell counts of IFNγ, TNFα and IL-2 producing Db-gp33 peptide stimulated Wt and *Hdac7^ko* CD8⁺ T cells over the course of $LCMV_{Arm}$ infection. **(J-L)** Cell counts of IFNγ, TNFα and IL-2 producing gp276 stimulated Wt and *Hdac7^ko* CD8⁺ T cells over the course of $LCMV_{Arm}$ infection. (n=5-8, multiple t test) *p<0.05, **p<0.01, ***p<0.001.

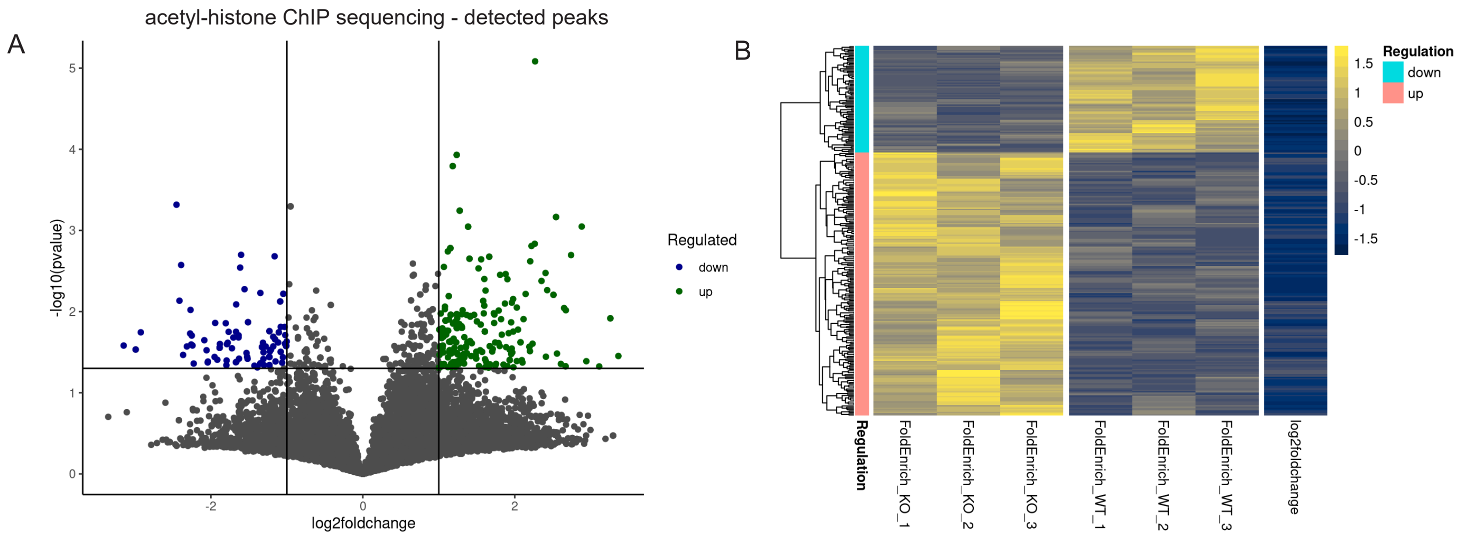

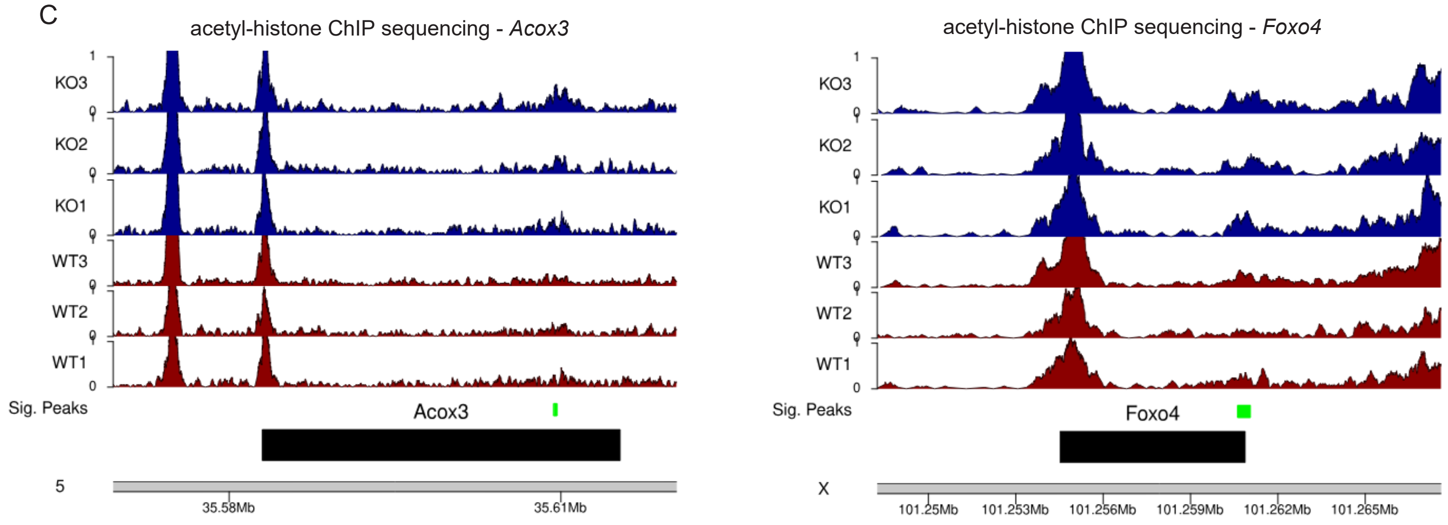

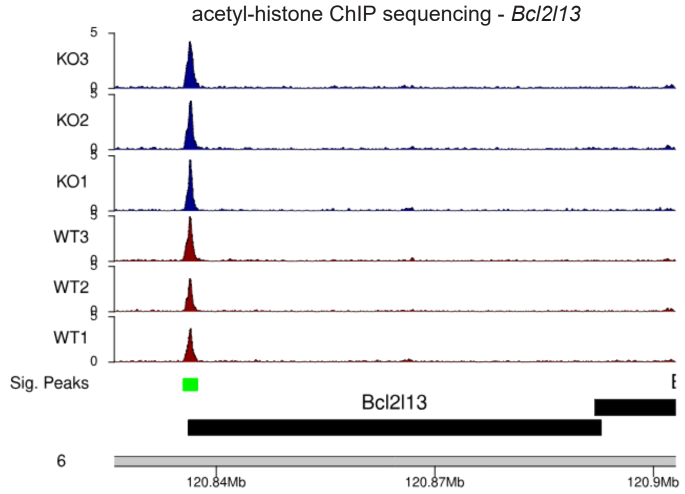

D

Gene expression CD8⁺ T cells *Hdac7ko* vs. Wt

Gene	Expression Value	Fold Change (log2)	p-Value	Adjusted p-Value (padj)
Acox3	57.81	2.82	4.78×10^{-13}	2.55×10^{-12}
Foxo4	47.00	3.11	1.46×10^{-12}	6.92×10^{-12}
Bcl2l13	134.83	2.41	1.23×10^{-17}	8.67×10^{-17}

Supplementary Figure 8: ChIP-seq show differences in histone acetylation between Hdac7^{ko} and Wt CD8^{+} T cells. (A) Chromatin-Immunoprecipitation (ChIP) was performed for Wt and *Hdac7^{ko}* CD8^{+} T cells for acetylated histone 3 (K9/K14). Volcano plot showing differentially acetylated gene sites, colors indicate statistical significance. **(B)** Peak enrichment of differentially detected acetylated histone 3 (K9/K14) peaks between Wt and *Hdac7^{ko}* CD8^{+} T cells. Fold enrichments of peaks for each sample were calculated relative to the IgG input. Values and fold change were provided by novogene, row wise scaled and visualized using ggplot2. **(C)** ChIP-seq tracks show differences in histone acetylation for *Acox3*, *Foxo4*, and *Bcl2l13* between *Hdac7^{ko}* and Wt. Read density was calculated and visualized using the “karyoplotR”-package for each sample. (Wt: red, *Hdac7^{ko}*: blue). Significantly different peaks are marked light green. (**D**) Genes upregulated in anti-CD3/CD28 activated, CD44^{+}CD62L^{+} naïve CD8^{+} *Hdac7^{ko}* T cells compared to the respective Wt.

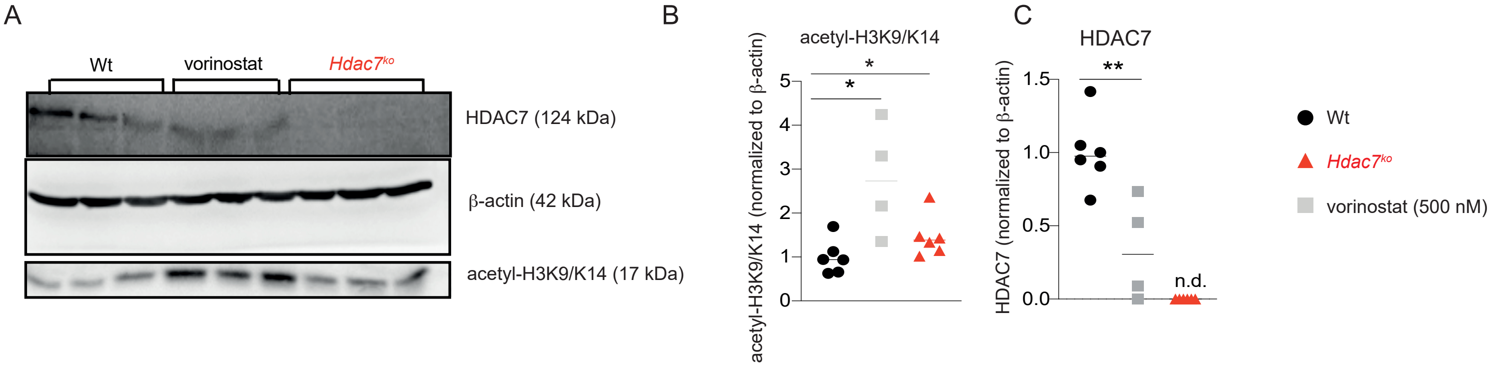

Supplementary Figure 9: Vorinostat treatment of Wt CD8⁺ T cells results in reduced HDAC7 protein expression. (A) Immunoblot analysis of HDAC7, acetylated-H3K9/K14 and β-actin in Wt CD8⁺ T cells activated with anti-CD3/CD28 antibodies and murine IL-2 for 48 h. Whole cell lysates were analyzed. Wt cells treated with vorinostat (500 nM) for 24 h served as positive control for acetylated-H3K9/K14 (representative of 3 independent experiments using biologically independent samples). **(B)** Fold-change protein expression of acetylated-H3K9/K14 and **(C)** HDAC7. Values were normalized to the intensity of β-actin bands (n=4-6, multiple t-test) *p<0.05, **p<0.01

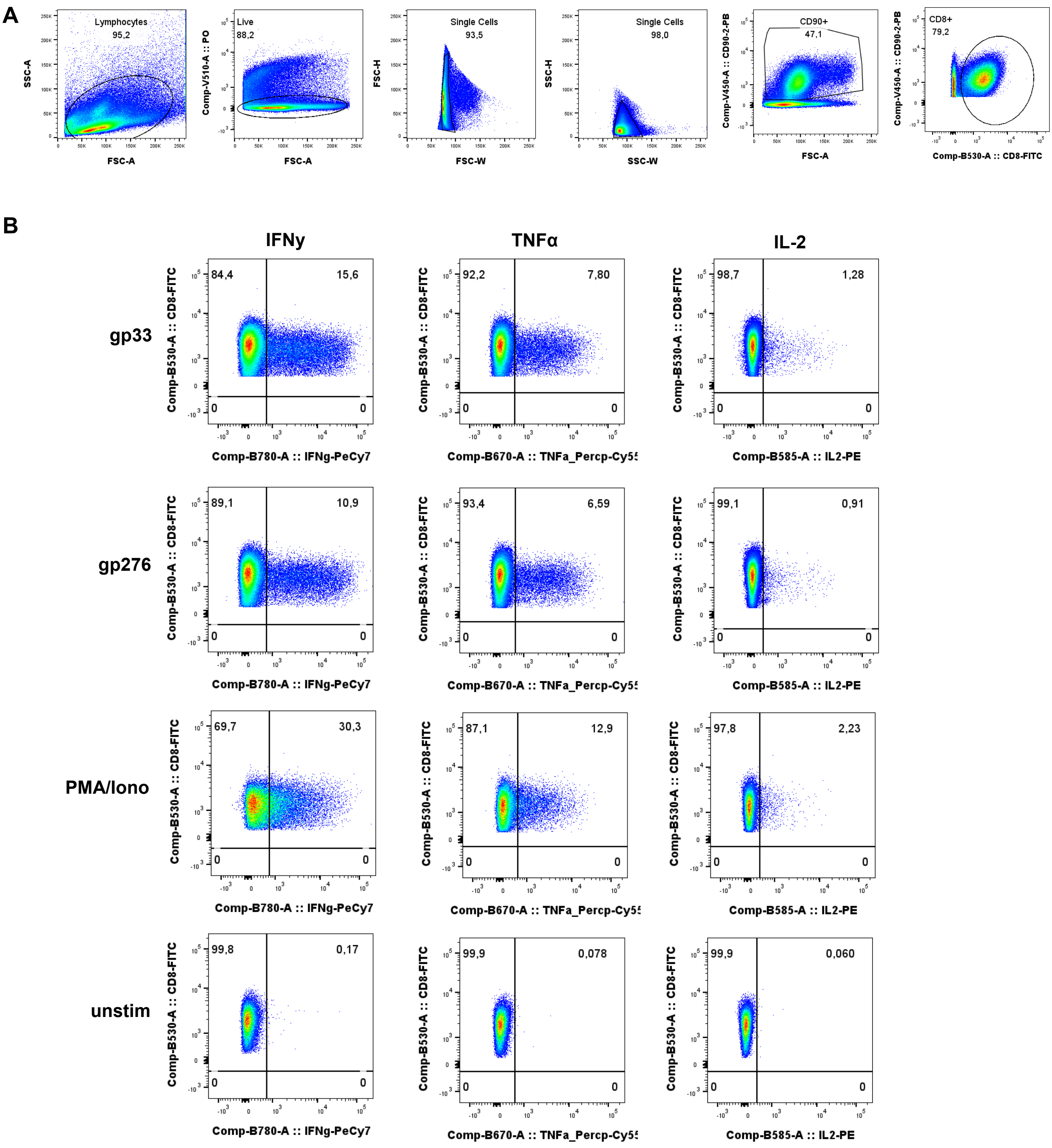

Supplementary Figure 10: Original cytometry plots for cytokine staining. (A) Gating strategy for the flow cytometric analysis of cytokine production of splenocytes from Wt mice on days 8 post $LCMV_{Arm}$ infection. **(B)** Representative pseudocolour flow cytometry plots of Wt splenocytes on day 8 post $LCMV_{Arm}$ infection. The plots illustrate staining for IFNγ, TNFα, and IL-2 following *in vitro* restimulation with gp33, gp276, or PMA/Ionomycin, as well as an unstimulated control treated with Brefeldin A only.
