# Supplementary material for: HDAC7 controls anti-viral and anti-tumor immunity by CD8+ T cells
Source: Front Immunol. 2026 May 12;17:1816695. doi: 10.3389/fimmu.2026.1816695 (PMC13201152; doi:10.3389/fimmu.2026.1816695)
Supplement: Supplementary file 2 [file Table1.pdf]

Supplementary Information

HDAC7 controls anti-viral and anti-tumor immunity by CD8$^+$ T cells.

Cansu Yerinde[1,2,§,#], Jacqueline Keye[1,2,4,#], Hsiang-Jung Hsiao[1,2], Sibel Durlanik[3], Inka Freise[1], Franziska Nowak[1,2], Marilena Letizia[1], Stephan Schlickeiser[3], Benedikt Obermayer[5], Adrian Huck[1,2], Marie Friedrich[1,2], Hao Wu[1,2], Désirée Kunkel[4], Anja A. Kühl[1,6], Sebastian Bauer[7,†], Andreas Thiel[3], Ahmed N. Hegazy[1,8,9], Britta Siegmund[1,10], Rainer Glauben[1,10,#,*] and Carl Weidinger[1,9,#]

[1] Charité – Universitätsmedizin Berlin, corporate member of Freie Universität Berlin, Humboldt-Universität zu Berlin; Medical Department, Division of Gastroenterology, Infectious Diseases and Rheumatology, Campus Benjamin Franklin, Berlin, Germany
[2] Freie Universität Berlin, Department of Chemistry, Biology, Pharmacy, Berlin, Germany
[3] Berlin Institute of Health at Charité - Universitätsmedizin Berlin, BIH Center for Regenerative Therapies, Berlin, Germany
[4] Berlin Institute of Health at Charité - Universitätsmedizin Berlin, Flow & Mass Cytometry Core Facility, Berlin, Germany
[5] Berlin Institute of Health at Charité – Universitätsmedizin Berlin, Core Unit Bioinformatics, Berlin, Germany
[6] Charité - Universitätsmedizin Berlin, iPATH. Berlin-Immunopathology for Experimental Models, Core Facility of the Charité, Berlin, Germany
[7] Berlin University of Applied Sciences, Berlin, Germany
[8] Deutsches Rheuma-Forschungszentrum, ein Institut der Leibniz-Gemeinschaft, Berlin, Germany
[9] Berlin Institute of Health at Charité – Universitätsmedizin Berlin, BIH Academy, Clinician Scientist Program
[10] German Cancer Consortium (DKTK), partner site Berlin; and German Cancer Research Center (DKFZ), Heidelberg, Germany

Current affiliations:

[$] Krantz Family Center for Cancer Research, Massachusetts General Hospital, Boston, MA, USA.

[#] Equal contribution

[†] Deceased

[*]*Corresponding author:*

Rainer Glauben, PhD

Charité - Universitätsmedizin Berlin, Medical Department, Division of Gastroenterology, Infectious Diseases and Rheumatology, Campus Benjamin Franklin

Hindenburgdamm 30, 12200 Berlin, Germany

phone: +49 30 450 614343, eMail: rainer.glauben@charite.de

Key words: LCMV, lymphoma, CD8$^+$ T cells, HDAC7, tumor-immunology, apoptosis, Store-operated calcium entry (SOCE), cellular exhaustion, Eomes, MEF2D, FASL, colitis

Supplementary table 1: Heavy metal isotope labeled antibodies for mass cytometry – Stimulation Panel

Heavy metal isotop	Antigen	Clone	Company	Catalog #	Dilution
141Pr	CD3e	145-2C11	Inhouse	Inhouse	1:100
142Nd	CD11c	N418	Fluidigm	3142003B	1:100
143Nd	IL-5	TRFK5	Fluidigm	3143003B	1:100
144Nd	IL-2	JES6-5H4	Fluidigm	3144002B	1:100
145Nd	CD69	H1.2F3	Fluidigm	3145005B	1:100
146Nd	IgM	X-54	Miltenyi Biotec	custom product	1:400
147Sm	CD45	30-F11	Fluidigm	3147003B	1:400
148Nd	CD11b	M1/70	Fluidigm	3148003B	1:200
149Sm	CD19	6D5	Fluidigm	3149002B	1:200
150Nd	CD138	REA104	Miltenyi Biotec	custom product	1:400
151Eu	CD25	3C7	Fluidigm	3151007B	1:200
152Sm	Ly-6G	REA526	Miltenyi Biotec	custom product	1:400
153Eu	Ly-6C	1G7.G10	Miltenyi Biotec	custom product	1:400
154Sm	TER-119	TER-119	Fluidigm	3154005B	1:200
155Gd	I-A/I-E	M5/114.15.2	Miltenyi Biotec	custom product	1:1000
156Gd	CD40L	MR1	Inhouse	Inhouse	1:100
158Gd	CD80	16-10A1	Miltenyi Biotec	custom product	1:200
159Tb	CD86	PO3.3	Miltenyi Biotec	custom product	1:200
160Gd	CD62L	MEL-14	Fluidigm	3160008B	1:100
161Dy	CD40	HM40-3	Biolegend	102908	1:200
162Dy	TNFα	MP6-XT22	Fluidigm	3162002B	1:100
163Dy	IgD	11-26c	Inhouse	Inhouse	1:2000
164Dy	CD83	REA304	Miltenyi Biotec	custom product	1:200
165Ho	IL-10	JES5-16E3	Inhouse	custom product	1:400
166Er	IL-4	11B11	Fluidigm	3166003B	1:100
167Er	IL-6	MP5-20F3	Fluidigm	3167003B	1:100
168Er	CD8	53-6.7	Inhouse	Inhouse	1:200
169Tm	TCRβ	H57-597	Fluidigm	3169002B	1:400
170Er	NK1.1	PK136	Fluidigm	3170002B	1:200
171Yb	CD44	IM7	Fluidigm	3171003B	1:400
172Yb	CD4	GK1.5	Inhouse	Inhouse	1:500
173Yb	IFNγ	XMG1.2	Inhouse	Inhouse	1:1000

174Yb	IL-17A	TC11-18H10.1	Fluidigm	3174002B	1:100
175Lu	CD38	90	Fluidigm	3175014B	1:400
176Yb	B220	RA3-6B2	Fluidigm	3176002B	1:400

Supplementary table 2: Heavy metal isotope labeled antibodies for mass cytometry – Surface Panel

Heavy metal isotop	Antigen	Clone	Company	Catalog #	Dilution
141Pr	CD3e	145-2C11	Inhouse	Inhouse	1:100
142Nd	CD11c	N418	Fluidigm	3142003B	1:100
143Nd	CD103	2E7	BioLegend	121402	1:200
144Nd	CD115	AFS98	Fluidigm	3144012B	1:100
145Nd	CD69	H1.2F3	Fluidigm	3145005B	1:100
146Nd	IgM	X-54	Miltenyi Biotec	custom product	1:400
147Sm	CD45	30-F11	Fluidigm	3147003B	1:400
148Nd	CD11b	M1/70	Fluidigm	3148003B	1:200
149Sm	CD19	6D5	Fluidigm	3149002B	1:200
150Nd	CD138	REA104	Miltenyi Biotec	custom product	1:400
151Eu	CD25	3C7	Fluidigm	3151007B	1:200
152Sm	Ly-6G	REA526	Miltenyi Biotec	custom product	1:400
153Eu	Ly-6C	1G7.G10	Miltenyi Biotec	custom product	1:400
154Sm	TER-119	TER-119	Fluidigm	3154005B	1:200
155Gd	I-A/I-E	M5/114.15.2	Miltenyi Biotec	custom product	1:1000
156Gd	PD-L1	10F.9G2	BioXCell	BE0101	1:400
158Gd	CD80	16-10A1	Miltenyi Biotec	custom product	1:200
159Tb	CD86	PO3.3	Miltenyi Biotec	custom product	1:200
160Gd	CD62L	MEL-14	Fluidigm	3160008B	1:100
161Dy	CD40	HM40-3	Biolegend	102908	1:200
163Dy	IgD	11-26c	Inhouse	Inhouse	1:2000
164Dy	CD83	REA304	Miltenyi Biotec	custom product	1:200
165Ho	PD-L2	TY25	BioLegend	107202	1:400
168Er	CD8	53-6.7	Inhouse	Inhouse	1:200
169Tm	TCRβ	H57-597	Fluidigm	3169002B	1:400
170Er	NK1.1	PK136	Fluidigm	3170002B	1:200
171Yb	CD44	IM7	Fluidigm	3171003B	1:400
172Yb	CD4	GK1.5	Inhouse	Inhouse	1:500
173Yb	F4/80	BM8	BioLegend	123143	1:100
175Lu	CD38	90	Fluidigm	3175014B	1:400
176Yb	B220	RA3-6B2	Fluidigm	3176002B	1:400

Supplementary table 3: Flow cytometry antibodies

Antigen	*Clone*	*Fluorochrome*	*Company*	*Catalog#*	*Dilution*
CD122	TM-b1	FITC	eBioscience	11-1222-81	1:200
CD127	A7R34	BV421	Biolegend	135023	1:200
CD127	A7R34	PerCP-Cy5.5	eBioscience	45-127182	1:100
CD178 (FasL)	MFL3	APC-eFluor780	eBioscience	47-5911-82	1:100
CD279 (PD-1)	J.43	eFluor450	eBioscience	48-9985-82	1:100
CD3	145-2C11	Alexa594	Inhouse		1:600
CD3	145-2C11	APC	eBioscience	17-0031-82	1:100
CD3	145-2C11	PerCpCy5	BD	551163	1:300
CD366 (Tim-3)	RMT3-23	PE-Cy7	Biolegend	119716	1:100
CD4	GK1.5	FITC	Miltenyi	130-091-608	1:50
CD4	GK1.5	FITC	BD	553729	1:200
CD4	GK1.5	APC-eFluor780	eBioscience	47-0041-80	1:300
CD40L	MR1	APC	Miltenyi	130-092-105	1:20
CD44	IM-7	Alexa700	Biolegend	103026	1:200
CD45.1	A20	APC-Cy7	Biolegend	110716	1:100
CD62L	MEL-14	APC-Cy7	eBioscience	104428	1:600
CD8	53-6.7	PerCp-eFluor710	eBioscience	46-0081-82	1:400
CD8	53-6.7	Pacific Blue	Biolegend	100725	1:100
CD8	53-6.7	FITC	Biolegend	100706	1:400
CD8	53-6.7	PE	Miltenyi	130-091-603	1:20
CD8	53-6.7	PerCp-Cy5	Biolegend	100733	1:200
CD8	53-6.7	APC	eBioscience	17-0081-81	1:500
CD8a	53-6.7	APC	eBioscience	17-0081-81	1:500
CD8a	53-6.7	FITC	Biolegend	100706	1:200
CD90.1 (Thy1.1)	OX-7	PE	Biolegend	202524	1:1000
CD90.2	53-2.1	eFluor650	eBioscience	95-0902-42	1:150
CD90.2	53-2.1	Alexa647	Biolegend	105318	1:800
CD90.2	53-2.1	Pacific Blue	Biolegend	140306	1:500
CD90.2 (Thy1.2)	30-H12	FITC	Biolegend	105306	1:200
Eomes	Dan11mag	Alexa488	eBioscience	53-4875-80	1:100
Eomes	Dan11mag	eFluor450	Invitrogen	48-4875-82	1:100
Fc-Rezeptor	24G2	--	Inhouse		1:400
FoxP3	FJK-16a	PE-Cy7	eBioscience	25-5773-82	1:100
IFNγ	XMG1.2	PE-Cy7	eBioscience	25-7311-82	1:500

IFNγ	XMG1.2	APC-Cy7	BD	561479	1:100
IFNγ	XMG1.2	APC	BD	554413	1:200
IFNγ	XMG1.2	APC	BD	554412	1:200
IL-17	TC11-18H10	Alexa488	BD	560221	1:50
IL-2	JES6-5H4	PE	Biolegend	503808	1:400
IL-4	11B11	PE	BD	554435	1:50
Ki-67	B56	PerCp-Cy5.5	BD	561284	1:200
KLRG1	2F1	APC	eBioscience	17-5893-82	1:150
KLRG1	2F1	APC-eFluor780	ebioscience	47-5893-80	1:100
PD-1	29F.1A12	PE-Cy7	Biolegend	135216	1:200
T-bet	4B10	PE-Cy7	Biolegend	644823	1:100
Tigit	GIGD7	FITC	eBioscience	11-9501-82	1:100
Tim-3	RMT3-23	PE-Cy7	Biolegend	25-5870-82	1:100
TNFα	MP6-XT22	PerCp-Cy5.5	Biolegend	506322	1:200
TNFα	MP6-XT22	PE-Cy7	BD	557644	1:200
CD178 (FasL)	MFL3	APC-eFluor780	eBioscience	47-5911-82	1:100
CD95 (Fas)	DX2	FITC	eBioscience	11-0959-42	1:100

Supplementary table 4: Western blot antibodies

Antibody anti-	***Host***	***Clone***	***Company***	***Dilution***
HDAC7	rabbit	D4E1L	Cell Signaling Technology	1:1000
β-actin	mouse	AC-74	Sigma-Aldrich	1:2000
acetyl-Histone 3-lysine 9/14	rabbit	polyclonal	Cell Signaling Technology	1:1000
MEF2D	rabbit	polyclonal	Abcam	1:1000<br>1:100 for IP

Supplementary table 5: Antibodies used in multiplex histology staining

Antibody anti-	***Clone***	***Company***	***Dilution***
CD8	D8A8Y	Cell Signaling Technology	1:500
Tim-3	D3M9R	Cell Signaling Technology	1:2000
PD-1	D7D5W	Cell Signaling Technology	1:300
Lag-3	polyclonal	Abcam	1:800
